# Supplementary material for: The risk of Plasmodium vivax parasitaemia after P. falciparum malaria: An individual patient data meta-analysis from the WorldWide Antimalarial Resistance Network
Source: PLoS Med. 2020 Nov 19;17(11):e1003393. doi: 10.1371/journal.pmed.1003393 (PMC7676739; doi:10.1371/journal.pmed.1003393)
Supplement: S1 Box — (PDF) [file pmed.1003393.s002.pdf]

S1 Box. Search strategy

Search strategy

As described previously,[7] prospective *P. falciparum* antimalarial clinical trials with a minimum of 28 days follow up, published between Jan 1, 1960 and Jan 5, 2018 in areas co-endemic for *P. falciparum* and *P. vivax* were identified by the application of the key terms (listed below) through Medline (Pubmed), Web of Science, Embase and the Cochrane Database of Systematic Reviews. Studies including treatment of uncomplicated *P. falciparum* mono-infection and mixed *P. falciparum* and *P. vivax* infection were included. Abstracts of all references containing any mention of antimalarial drugs were manually checked to confirm prospective clinical trials, with review of full text when needed. Studies on prevention, prophylaxis, reviews, animal studies, patients with severe malaria, where schizontocidal treatment was unsupervised or where data were extracted retrospectively from medical records outside of a planned trial were excluded.

In the current study, a subset of studies were identified by restricting the inclusion criteria to studies in which patients were treated with one of four ACTs: artemether-lumefantrine, dihydroartemisinin-piperaquine, artesunate-mefloquine, or artesunate-amodiaquine. Studies were excluded if ACTs were given with adjunctive treatments, *P. vivax* was not reported during follow up, patients were all hyperparasitaemic or studies enrolled patients infected with malaria from diverse locations such as travellers or soldiers.

Key terms

Literature search (conducted January 2018) with the following key terms (version undertaken in Pubmed): (malaria OR plasmod*) AND (amodiaquine OR atovaquone OR artemisinin OR arteether OR artesunate OR artemether OR artemether OR artemotil OR azithromycin OR artekin OR chloroquine OR chlorproguanil OR cycloguanil OR clindamycin OR coartem OR dapsone OR dihydroartemisinin OR duo-cotecxin OR doxycycline OR halofantrine OR lumefantrine OR lariam OR malarone OR mefloquine OR naphthoquine OR naphthoquinone OR piperaquine OR primaquine OR proguanil OR pyrimethamine OR pyronaridine OR proguanil OR quinidine OR quinine OR riamet OR sulphadoxine OR tetracycline OR tafenoquine).
