# Supplementary material for: The risk of Plasmodium vivax parasitaemia after P. falciparum malaria: An individual patient data meta-analysis from the WorldWide Antimalarial Resistance Network
Source: PLoS Med. 2020 Nov 19;17(11):e1003393. doi: 10.1371/journal.pmed.1003393 (PMC7676739; doi:10.1371/journal.pmed.1003393)
Supplement: S1 Text — (PDF) [file pmed.1003393.s003.pdf]

S1 Text. Assessment of risk of bias relating to individual studies

The Joanne Briggs Institute Critical Appraisal Tool for Systematic Reviews of Prevalence Studies[18] was used to assess bias for each study within nine domains: 1) Sample frame, 2) Recruitment, 3) Sample size, 4) Subject and setting description, 5) Sufficient coverage, 6) Method of identification, 7) Method reliability, 8) Analysis, and 9) Response rate. Risk of bias was assessed for the prevalence of recurrent *P. vivax* and *P. falciparum* parasitaemia rather than the study itself. Risk of bias was assessed by two independent reviewers.

Risk of bias assessment – Minimum criteria for low risk

Domain	Minimum criteria for low risk	Comments
1. Sample frame	Male and female patients, and children and adults included. No other significant bias in inclusion criteria	
2. Recruitment	Site of recruitment (eg hospital, clinic) described and did not exclude specific groups (eg drug sensitive patients)	
3. Sample size	Sample size was adequate (considered to be >138 patients in each treatment arm).	Derived using the formula by Naing *et al* described in the Joanne Briggs Institute tool.[18, 196] *P. vivax* parasitaemia assumed to be 10%.
4. Subject and setting description	Setting and subjects described including age, gender, baseline parasitaemia and presence of mixed infection.	
5. Sufficient coverage (Group comparison)	Loss to follow up similar between treatment arms or not related to outcome.	
6. Method of identification	Microscopy used to identify recurrent parasitaemia.	Microscopy was used in every study
7. Method reliability	Microscopy used to identify recurrent parasitaemia.	Microscopy was used in every study
8. Analysis	Not applicable as individual patient data provided.	
9. Response rate	<10% of enrolled patients lost to follow up or reasons for lost to follow up explained and unrelated to recurrent parasitaemia.	

References

18. Munn Z, Moola S, Lisy K, Riitano D, Tufanaru C. Methodological guidance for systematic reviews of observational epidemiological studies reporting prevalence and cumulative incidence data. Int J Evid Based Healthc. 2015;13(3):147-53. Epub 2015/09/01. doi: 10.1097/XEB.0000000000000054. PubMed PMID: 26317388.

196. Naing L, Winn T, Rusli BN. Practical issues in calculating the sample size for prevalence studies. Archives of Orofacial Sciences. 2006;1:9-14.

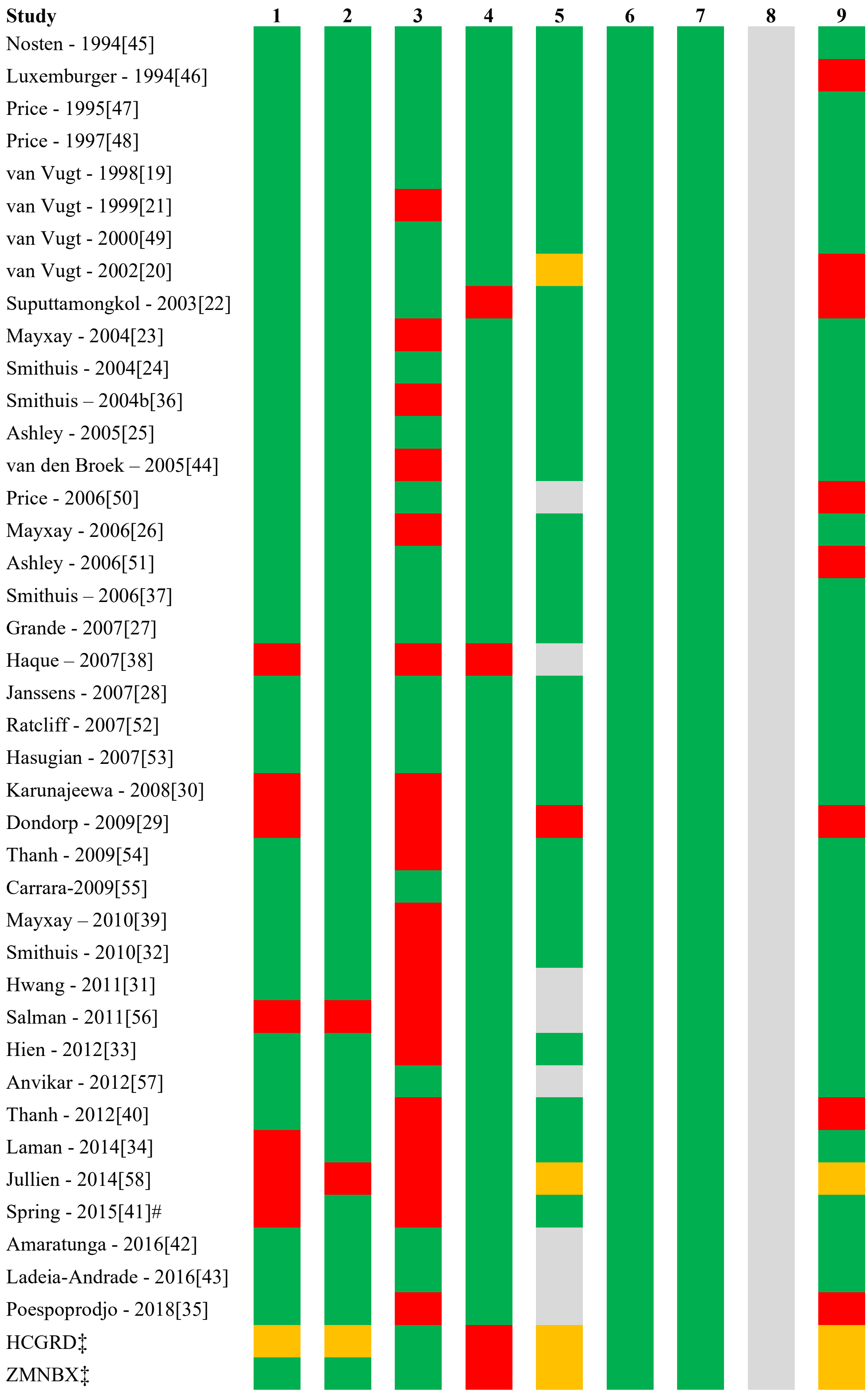

Risk of bias assessment: Red – High risk; Orange – Unknown; Green – Low risk; Grey – not applicable; Categories of bias 1-9 described in the table above.
