# Supplementary figures and images for: The risk of Plasmodium vivax parasitaemia after P. falciparum malaria: An individual patient data meta-analysis from the WorldWide Antimalarial Resistance Network
Source: PLoS Med. 2020 Nov 19;17(11):e1003393. doi: 10.1371/journal.pmed.1003393 (PMC7676739; doi:10.1371/journal.pmed.1003393)

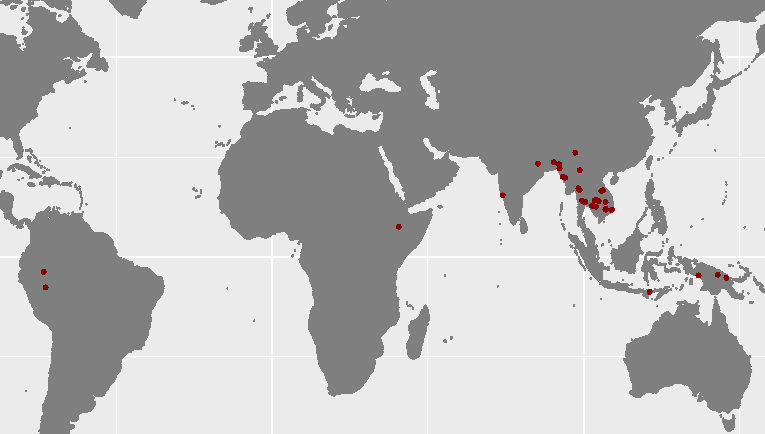

Supplement: S1 Fig — Map created using ggplot2 in R. (TIF) [file pmed.1003393.s004.tif]

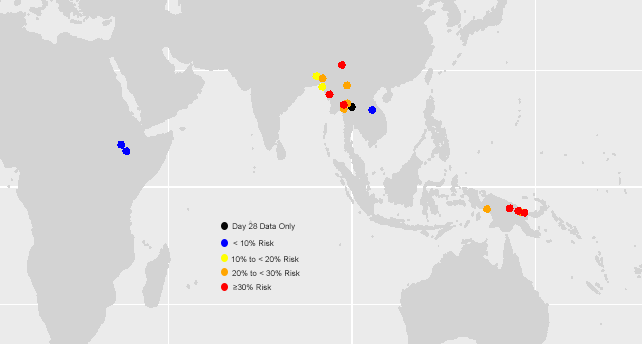

Supplement: S2 Fig — Map created using ggplot2 in R. AL, artemether-lumefantrine (TIF) [file pmed.1003393.s005.tif]
