# Supplementary material for: The risk of Plasmodium vivax parasitaemia after P. falciparum malaria: An individual patient data meta-analysis from the WorldWide Antimalarial Resistance Network
Source: PLoS Med. 2020 Nov 19;17(11):e1003393. doi: 10.1371/journal.pmed.1003393 (PMC7676739; doi:10.1371/journal.pmed.1003393)
Supplement: S3 Fig — Risk of P. vivax parasitaemia at day 42 following treatment with AA according to subnational (A) P. vivax incidence and (B) P. falciparum incidence. AA, artesunate-amodiaquine (PDF) [file pmed.1003393.s006.pdf]

S3 Figure. Risk of *P. vivax* parasitaemia at day 42 following treatment with artesunate-amodiaquine according to background subnational incidence of *P. vivax* (A) and *P. falciparum* (B).

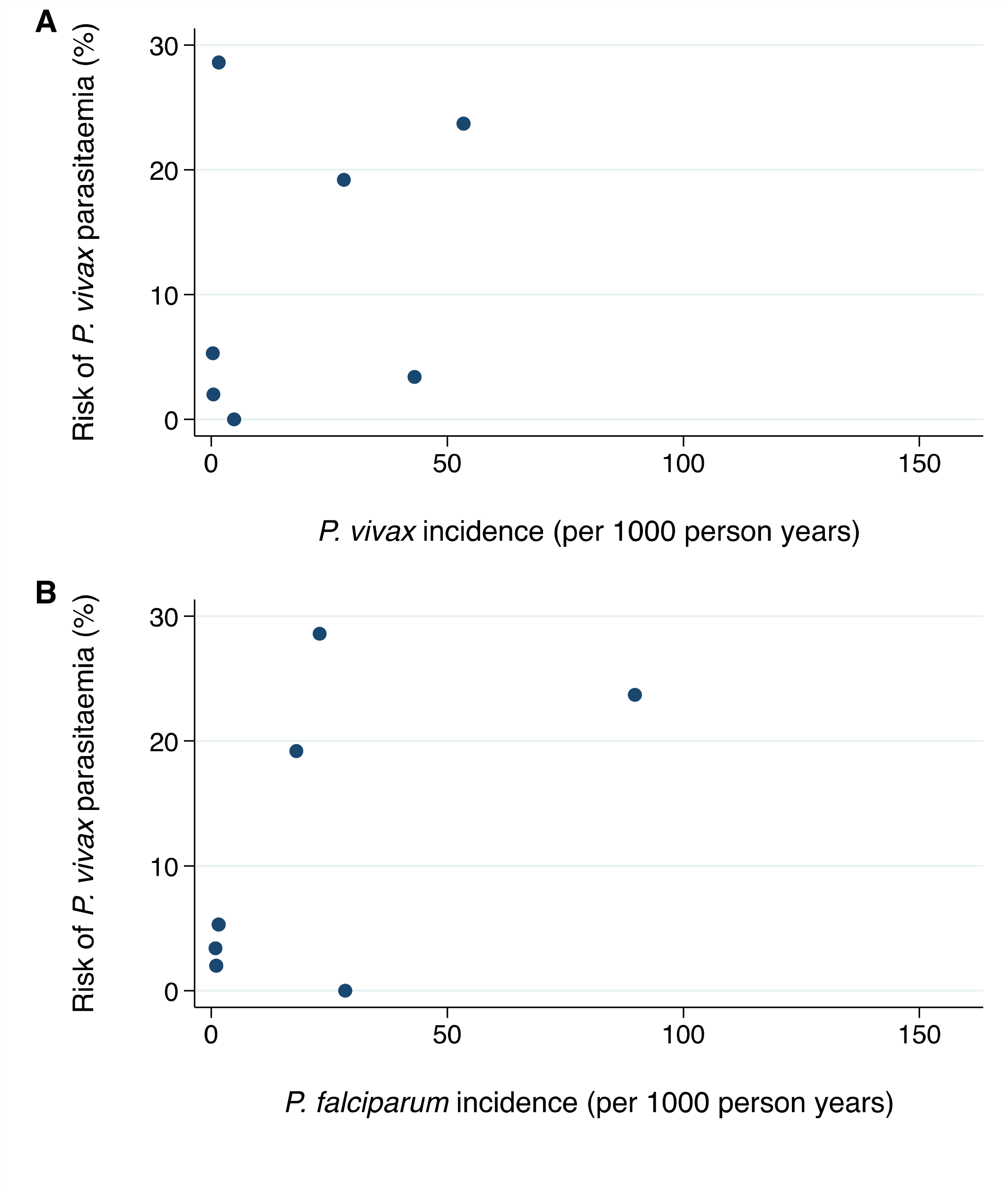

Unadjusted r_s=0.285, p=0.5352 (A) and r_s=0.557, p=0.1942 (B)
