# Supplementary material for: The risk of Plasmodium vivax parasitaemia after P. falciparum malaria: An individual patient data meta-analysis from the WorldWide Antimalarial Resistance Network
Source: PLoS Med. 2020 Nov 19;17(11):e1003393. doi: 10.1371/journal.pmed.1003393 (PMC7676739; doi:10.1371/journal.pmed.1003393)
Supplement: S4 Fig — Risk of P. vivax parasitaemia at day 42 following treatment with AM according to subnational (A) P. vivax incidence and (B) P. falciparum incidence. AM, artesunate-mefloquine (PDF) [file pmed.1003393.s007.pdf]

S4 Figure. Risk of *P. vivax* parasitaemia at day 42 following treatment with artesunate-mefloquine according to the background subnational incidence of *P. vivax* (A) and *P. falciparum* (B).

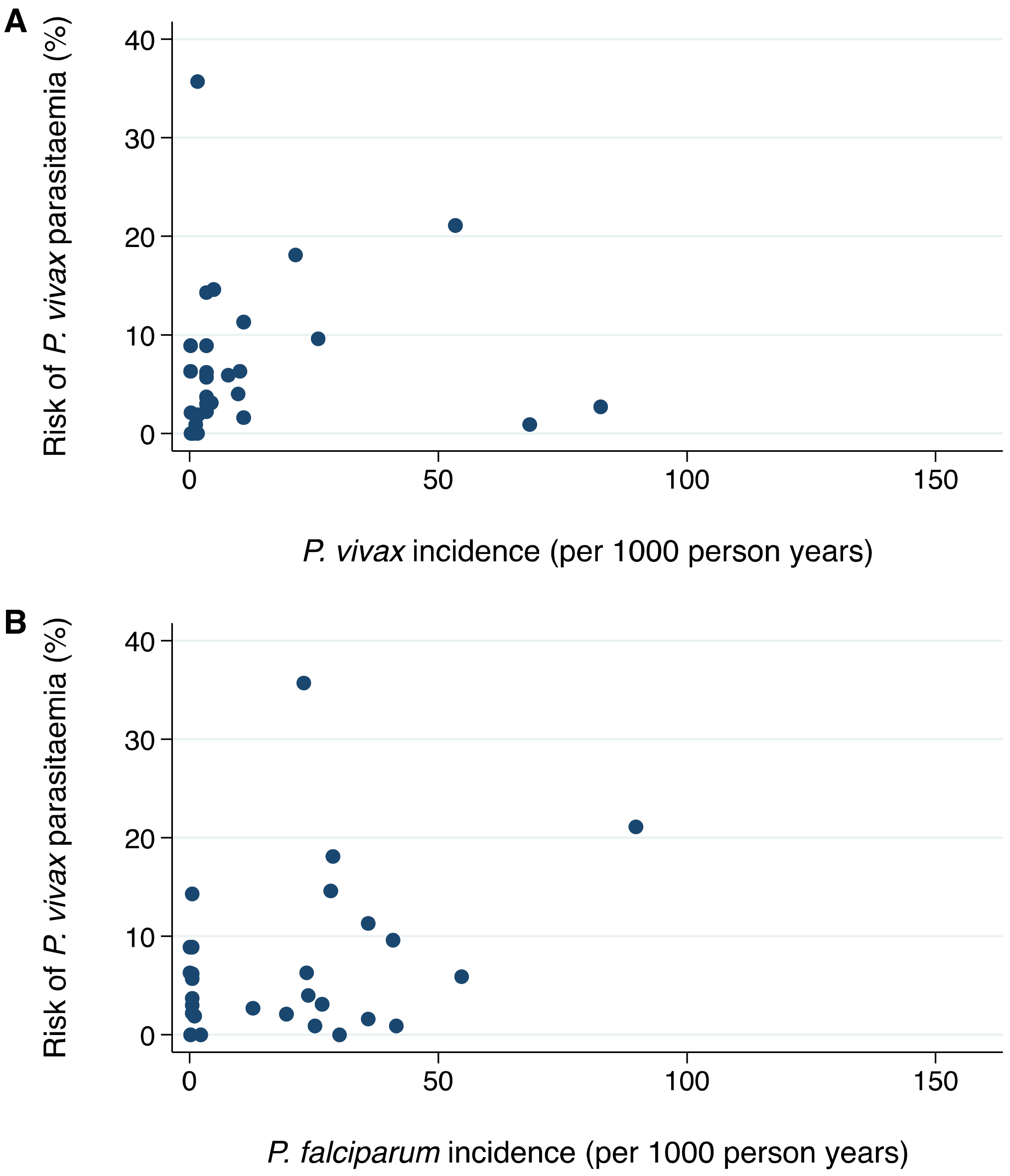

Unadjusted r_s=0.045, p=0.8187 (A) and r_s=0.311, p=0.1070 (B)
