# Supplementary material for: The risk of Plasmodium vivax parasitaemia after P. falciparum malaria: An individual patient data meta-analysis from the WorldWide Antimalarial Resistance Network
Source: PLoS Med. 2020 Nov 19;17(11):e1003393. doi: 10.1371/journal.pmed.1003393 (PMC7676739; doi:10.1371/journal.pmed.1003393)
Supplement: S1 Table — (PDF) [file pmed.1003393.s009.pdf]

S1 Table. Reasons for studies not being included in analysis

Reason	Number of studies	Studies*
No eligible ACT	92	[69-88]
		[89-108]
		[109-128]
		[129-148]
		[149-160]
P. vivax not reported on follow up	10	[161-170]
Data not available	23	[171-187]
		[188-193]
Essential data not available	2	[194-195]

Studies described were identified in a prior systematic review.[7]

References

7. Commons RJ, Simpson JA, Thriemer K, Hossain MS, Douglas NM, Humphreys GS, et al. Risk of Plasmodium vivax parasitaemia after Plasmodium falciparum infection: a systematic review and meta-analysis. Lancet Infect Dis. 2019;19(1):91-101. Epub 2018/12/28. doi: 10.1016/S1473-3099(18)30596-6. PubMed PMID: 30587297.

69. Segal HE, Chinvanthananond P, Laixuthai B, Phintuyothin P, Pearlman EJ, Na-Nakorn A, et al. Preliminary study of WR 33063 in the treatment of falciparum malaria in northeast Thailand. Am J Trop Med Hyg. 1974;23(4):560-4. Epub 1974/07/01. PubMed PMID: 4603134.

70. Hall AP, Segal HE, Pearlman EJ, Phintuyothin P, Kosakal S. Amodiaquine resistant falciparum malaria in Thailand. Am J Trop Med Hyg. 1975;24(4):575-80. Epub 1975/07/01. PubMed PMID: 1098499.

71. Segal HE, Chinvanthananond P, Laixuthai B, Pearlman EJ, Hall AP, Phintuyothin P, et al. Comparison of diaminodiphenylsulphonepyrimethamine and sulfadoxine-pyrimethamine combinations in the treatment of falciparum malaria in Thailand. Trans R Soc Trop Med Hyg. 1975;69(1):139-42. Epub 1975/01/01. PubMed PMID: 1096374.

72. Doberstyn EB, Phintuyothin P, Noeypatimanondh S, Teerakiartkamjorn C. Single-dose therapy of falciparum malaria with mefloquine or pyrimethamine-sulfadoxine. Bull World Health Organ. 1979;57(2):275-9. Epub 1979/01/01. PubMed PMID: 373903; PubMed Central PMCID: PMCPMC2395771.

73. de Souza JM. A phase II clinical trial of mefloquine in Brazilian male subjects. Bull World Health Organ. 1983;61(5):815-20. Epub 1983/01/01. PubMed PMID: 6360401; PubMed Central PMCID: PMCPMC2536166.

74. Harinasuta T, Bunnag D, Wernsdorfer WH. A phase II clinical trial of mefloquine in patients with chloroquine-resistant falciparum malaria in Thailand. Bull World Health Organ. 1983;61(2):299-305. Epub 1983/01/01. PubMed PMID: 6345013; PubMed Central PMCID: PMCPMC2536127.

75. Li GQ, Arnold K, Guo XB, Jian HX, Fu LC. Randomised comparative study of mefloquine, qinghaosu, and pyrimethamine-sulfadoxine in patients with falciparum malaria. Lancet. 1984;2(8416):1360-1. Epub 1984/12/15. PubMed PMID: 6150365.

76. Botero D, Restrepo M, Montoya A. Prospective double-blind trial of two different doses of mefloquine plus pyrimethamine-sulfadoxine compared with pyrimethamine-sulfadoxine alone in the treatment of falciparum malaria.

Bull World Health Organ. 1985;63(4):731-7. Epub 1985/01/01. PubMed PMID: 3910296; PubMed Central PMCID: PMCPMC2536382.

77. de Souza JM, Sheth UK, de Oliveira RM, Roulet H, de Souza SD. An open, randomized, phase III clinical trial of mefloquine and of quinine plus sulfadoxine-pyrimethamine in the treatment of symptomatic falciparum malaria in Brazil. Bull World Health Organ. 1985;63(3):603-9. Epub 1985/01/01. PubMed PMID: 3899397; PubMed Central PMCID: PMCPMC2536431.

78. Harinasuta T, Bunnag D, Lasserre R, Leimer R, Vinijanont S. Trials of mefloquine in vivax and of mefloquine plus 'fansidar' in falciparum malaria. Lancet. 1985;1(8434):885-8. Epub 1985/04/20. PubMed PMID: 2858743.

79. Meek SR, Doberstyn EB, Gauzere BA, Thanapanich C, Nordlander E, Phuphaisan S. Treatment of falciparum malaria with quinne and tetracycline or combined mefloquine/sulfadoxine/pyrimethamine on the Thai-Kampuchean border. Am J Trop Med Hyg. 1986;35(2):246-50. Epub 1986/03/01. PubMed PMID: 3513642.

80. Pe Than M, Tin S. The efficacy of artemether (qinghaosu) in *Plasmodium falciparum* and *P. vivax* in Burma. Southeast Asian J Trop Med Public Health. 1986;17(1):19-22. Epub 1986/03/01. PubMed PMID: 3526578.

81. Chongsuphajaisiddhi T, Sabchareon A, Chantavanich P, Singhasivanon V, Attanath P, Wernsdorfer WH, et al. A phase-III clinical trial of mefloquine in children with chloroquine-resistant falciparum malaria in Thailand. Bull World Health Organ. 1987;65(2):223-6. Epub 1987/01/01. PubMed PMID: 3301042; PubMed Central PMCID: PMCPMC2490838.

82. Harinasuta T, Bunnag D, Vanijanond S, Charoenlarp P, Suntharasmai P, Chitamas S, et al. Mefloquine, sulfadoxine, and pyrimethamine in the treatment of symptomatic falciparum malaria: a double-blind trial for determining the most effective dose. Bull World Health Organ. 1987;65(3):363-7. Epub 1987/01/01. PubMed PMID: 3311439; PubMed Central PMCID: PMCPMC2490999.

83. Nosten F, Imvithaya S, Vincenti M, Delmas G, Lebihan G, Hausler B, et al. Malaria on the Thai-Burmese border: treatment of 5192 patients with mefloquine-sulfadoxine-pyrimethamine. Bull World Health Organ. 1987;65(6):891-6. Epub 1987/01/01. PubMed PMID: 3325187; PubMed Central PMCID: PMCPMC2491097.

84. Boudreau EF, Pang LW, Dixon KE, Webster HK, Pavanand K, Tosingha L, et al. Malaria: treatment efficacy of halofantrine (WR 171,669) in initial field trials in Thailand. Bull World Health Organ. 1988;66(2):227-35. Epub 1988/01/01. PubMed PMID: 3293828; PubMed Central PMCID: PMCPMC2491042.

85. Kremsner PG, Zotter GM, Graninger W, Rocha RM, Bienzle U, Feldmeier H. Clindamycin is effective against *Plasmodium falciparum* but not against *P. vivax* in mixed infections. Trans R Soc Trop Med Hyg. 1989;83(3):332-3. Epub 1989/05/01. PubMed PMID: 2694469.

86. Salazar NP, Saniel MC, Estoque MH, Talao FA, Bustos DG, Palogan LP, et al. Oral clindamycin in the treatment of acute uncomplicated falciparum malaria. Southeast Asian J Trop Med Public Health. 1990;21(3):397-403. Epub 1990/09/01. PubMed PMID: 2075481.

87. Bunnag D, Viravan C, Looareesuwan S, Karbwang J, Harinasuta T. Double blind randomised clinical trial of oral artesunate at once or twice daily dose in falciparum malaria. Southeast Asian J Trop Med Public Health. 1991;22(4):539-43. Epub 1991/12/01. PubMed PMID: 1820641.

88. Bunnag D, Viravan C, Looareesuwan S, Karbwang J, Harinasuta T. Double blind randomised clinical trial of two different regimens of oral artesunate in falciparum malaria. Southeast Asian J Trop Med Public Health. 1991;22(4):534-8. Epub 1991/12/01. PubMed PMID: 1820640.

89. Bunnag D, Viravan C, Looareesuwan S, Karbwang J, Harinasuta T. Clinical trial of artesunate and artemether on multidrug resistant falciparum malaria in Thailand. A preliminary report. Southeast Asian J Trop Med Public Health. 1991;22(3):380-5. Epub 1991/09/01. PubMed PMID: 1818389.

90. Bunnag D, Karbwang J, Viravan C, Chitamas S, Harinasuta T. Clinical trials of mefloquine with tetracycline. Southeast Asian J Trop Med Public Health. 1992;23(3):377-82. Epub 1992/09/01. PubMed PMID: 1488688.

91. Bunnag D, Karbwang J, Harinasuta T. Artemether in the treatment of multiple drug resistant falciparum malaria. Southeast Asian J Trop Med Public Health. 1992;23(4):762-7. Epub 1992/12/01. PubMed PMID: 1298086.

92. Karbwang J, Bangchang KN, Thanavibul A, Bunnag D, Chongsuphajaisiddhi T, Harinasuta T. Comparison of oral artemether and mefloquine in acute uncomplicated falciparum malaria. Lancet. 1992;340(8830):1245-8. PubMed PMID: 1359318.

93. Karbwang J, Na-Bangchang K, Thanavibul A, Bunnag D, Chongsuphajaisiddhi T, Harinasuta T. Comparison of oral artesunate and quinine plus tetracycline in acute uncomplicated falciparum malaria. Bull World Health Organ. 1994;72(2):233-8. Epub 1994/01/01. PubMed PMID: 8205643; PubMed Central PMCID: PMCPMC2486535.

94. Looareesuwan S, Viravan C, Vanijanonta S, Wilairatana P, Charoenlarp P, Canfield CJ, et al. Randomized trial of mefloquine-doxycycline, and artesunate-doxycycline for treatment of acute uncomplicated falciparum malaria. Am J Trop Med Hyg. 1994;50(6):784-9. Epub 1994/06/01. PubMed PMID: 8024075.

95. Na Bangchang K, Karbwang J, Thomas CG, Thanavibul A, Sukontason K, Ward SA, et al. Pharmacokinetics of artemether after oral administration to healthy Thai males and patients with acute, uncomplicated falciparum malaria. Br J Clin Pharmacol. 1994;37(3):249-53. Epub 1994/03/01. PubMed PMID: 8198933; PubMed Central PMCID: PMCPMC1364755.

96. Oemijati S, Pribadi W, Wartati K, Arbani P, Suprijanto S, Rasisi R. Treatment of chloroquine-resistant *Plasmodium falciparum* infections with clindamycin hydrochloride in Dili, East Timor, Indonesia. Curr Ther Res Clin Exp. 1994;55(4):468-79.

97. Bunnag D, Kanda T, Karbwang J, Thimasarn K, Pungpak S, Harinasuta T. Artemether-mefloquine combination in multidrug resistant falciparum malaria. Trans R Soc Trop Med Hyg. 1995;89(2):213-5. Epub 1995/03/01. PubMed PMID: 7778152.

98. Karbwang J, Na-Bangchang K, Thanavibul A, Laothavorn P, Ditta-in M, Harinasuta T. A comparative clinical trial of artemether and the sequential regimen of artemether-mefloquine in multidrug resistant falciparum malaria. J Antimicrob Chemother. 1995;36(6):1079-83. Epub 1995/12/01. PubMed PMID: 8821610.

99. Karbwang J, Na-Bangchang K, Thanavibul A, Ditta-in M, Harinasuta T. A comparative clinical trial of two different regimens of artemether plus mefloquine in multidrug resistant falciparum malaria. Trans R Soc Trop Med Hyg. 1995;89(3):296-8. Epub 1995/05/01. PubMed PMID: 7660440.

100. Baird JK, Sismadi P, Masbar S, Leksana B, Sekartuti, Ramzan A, et al. Chloroquine sensitive *Plasmodium falciparum* and *P. vivax* in central Java, Indonesia. Trans R Soc Trop Med Hyg. 1996;90(4):412-3. Epub 1996/07/01. PubMed PMID: 8882191.

101. Bunnag D, Karbwang J, Na-Bangchang K, Thanavibul A, Chittamas S, Harinasuta T. Quinine-tetracycline for multidrug resistant falciparum malaria. Southeast Asian J Trop Med Public Health. 1996;27(1):15-8. Epub 1996/03/01. PubMed PMID: 9031393.

102. Duarte EC, Fontes CJ, Gyorkos TW, Abrahamowicz M. Randomized controlled trial of artesunate plus tetracycline versus standard treatment (quinine plus tetracycline) for uncomplicated *Plasmodium falciparum* malaria in Brazil. Am J Trop Med Hyg. 1996;54(2):197-202. Epub 1996/02/01. PubMed PMID: 8619447.

103. Looareesuwan S, Wilairatana P, Vanijanonta S, Pitisuttithum P, Viravan C, Kraisintu K. Treatment of acute, uncomplicated, falciparum malaria with oral dihydroartemisinin. Ann Trop Med Parasitol. 1996;90(1):21-8. Epub 1996/02/01. PubMed PMID: 8729624.

104. Looareesuwan S, Viravan C, Webster HK, Kyle DE, Hutchinson DB, Canfield CJ. Clinical studies of atovaquone, alone or in combination with other antimalarial drugs, for treatment of acute uncomplicated malaria in Thailand. Am J Trop Med Hyg. 1996;54(1):62-6. Epub 1996/01/01. PubMed PMID: 8651372.

105. Looareesuwan S, Kyle DE, Viravan C, Vanijanonta S, Wilairatana P, Wernsdorfer WH. Clinical study of pyronaridine for the treatment of acute uncomplicated falciparum malaria in Thailand. Am J Trop Med Hyg. 1996;54(2):205-9. Epub 1996/02/01. PubMed PMID: 8619449.

106. Mohapatra PK, Khan AM, Prakash A, Mahanta J, Srivastava VK. Effect of arteether alpha/beta on uncomplicated falciparum malaria cases in Upper Assam. Indian J Med Res. 1996;104:284-7. Epub 1996/11/01. PubMed PMID: 8979518.

107. Na-Bangchang K, Tipwangso P, Thanavibul A, Tan-ariya P, Suprakob K, Kanda T, et al. Artemether-pyrimethamine in the treatment of pyrimethamine-resistant falciparum malaria. Southeast Asian J Trop Med Public Health. 1996;27(1):19-23. Epub 1996/03/01. PubMed PMID: 9031394.

108. Na-Bangchang K, Kanda T, Tipawangso P, Thanavibul A, Suprakob K, Ibrahim M, et al. Activity of artemether-azithromycin versus artemether-doxycycline in the treatment of multiple drug resistant falciparum malaria. Southeast Asian J Trop Med Public Health. 1996;27(3):522-5. Epub 1996/09/01. PubMed PMID: 9185262.

109. Restrepo M, Botero D, Marquez RE, Boudreau EF, Navaratnam V. A clinical trial with halofantrine on patients with falciparum malaria in Colombia. Bull World Health Organ. 1996;74(6):591-7. Epub 1996/01/01. PubMed PMID: 9060219; PubMed Central PMCID: PMCPMC2486805.

110. de Alencar FE, Cerutti C, Jr., Durlacher RR, Boulos M, Alves FP, Milhous W, et al. Atovaquone and proguanil for the treatment of malaria in Brazil. J Infect Dis. 1997;175(6):1544-7. Epub 1997/06/01. PubMed PMID: 9180204.

111. Looareesuwan S, Wilairatana P, Viravan C, Vanijanonta S, Pitisuttithum P, Kyle DE. Open randomized trial of oral artemether alone and a sequential combination with mefloquine for acute uncomplicated falciparum malaria. Am J Trop Med Hyg. 1997;56(6):613-7. Epub 1997/06/01. PubMed PMID: 9230790.

112. Looareesuwan S, Wilairatana P, Vanijanonta S, Pitisuttithum P, Ratanapong Y, Andrial M. Monotherapy with sodium artesunate for uncomplicated falciparum malaria in Thailand: a comparison of 5- and 7-day regimens. Acta Trop. 1997;67(3):197-205. Epub 1997/09/30. PubMed PMID: 9241384.

113. Na-Bangchang K, Congpuong K, Sirichaisinthop J, Suprakorb K, Karbwang J. Compliance with a 2 day course of artemether-mefloquine in an area of highly multi-drug resistant *Plasmodium falciparum* malaria. Br J Clin Pharmacol. 1997;43(6):639-42. Epub 1997/06/01. PubMed PMID: 9205825; PubMed Central PMCID: PMCPMC2042779.

114. Karbwang J, Na-Bangchang K, Congpuong K, Thanavibul A, Wattanakoon Y, Molunto P. Pharmacokinetics of oral artemether in Thai patients with uncomplicated falciparum malaria. Fundam Clin Pharmacol. 1998;12(2):242-4. Epub 1998/05/05. PubMed PMID: 9565781.

115. Sabchareon A, Attanath P, Phanuaksook P, Chanthavanich P, Poonpanich Y, Mookmanee D, et al. Efficacy and pharmacokinetics of atovaquone and proguanil in children with multidrug-resistant *Plasmodium falciparum* malaria. Trans R Soc Trop Med Hyg. 1998;92(2):201-6. Epub 1998/10/09. PubMed PMID: 9764334.

116. Cerutti C, Jr., Durlacher RR, de Alencar FE, Segurado AA, Pang LW. In vivo efficacy of mefloquine for the treatment of falciparum malaria in Brazil. J Infect Dis. 1999;180(6):2077-80. Epub 1999/11/24. doi: 10.1086/315141. PubMed PMID: 10558975.

117. Looareesuwan S, Wilairatana P, Chalermarut K, Rattanapong Y, Canfield CJ, Hutchinson DB. Efficacy and safety of atovaquone/proguanil compared with mefloquine for treatment of acute *Plasmodium falciparum* malaria in Thailand. Am J Trop Med Hyg. 1999;60(4):526-32. Epub 1999/05/29. PubMed PMID: 10348224.

118. Na-Bangchang K, Tippanangkosol P, Ubalee R, Chaovanakawee S, Saenglertsilapachai S, Karbwang J. Comparative clinical trial of four regimens of dihydroartemisinin-mefloquine in multidrug-resistant falciparum malaria. Trop Med Int Health. 1999;4(9):602-10. Epub 1999/10/30. PubMed PMID: 10540300.

119. Pukrittayakamee S, Chantra A, Vanijanonta S, Clemens R, Looareesuwan S, White NJ. Therapeutic responses to quinine and clindamycin in multidrug-resistant falciparum malaria. Antimicrob Agents Chemother. 2000;44(9):2395-8. Epub 2000/08/22. PubMed PMID: 10952585; PubMed Central PMCID: PMCPMC90075.

120. Krudsood S, Singhasivanon P, Silachamroon U, Treeprasertsuk S, Kaivipakbanyai W, Chalermrut K, et al. Clinical trial of halofantrine with modified doses for treatment of malaria in the hospital for tropical diseases. Southeast Asian J Trop Med Public Health. 2001;32(2):255-61. Epub 2001/09/15. PubMed PMID: 11556573.

121. Tjitra E, Suprianto S, Currie BJ, Morris PS, Saunders JR, Anstey NM. Therapy of uncomplicated falciparum malaria: a randomized trial comparing artesunate plus sulfadoxine-pyrimethamine versus sulfadoxine-pyrimethamine alone in Irian Jaya, Indonesia. Am J Trop Med Hyg. 2001;65(4):309-17. Epub 2001/11/06. PubMed PMID: 11693875.

122. Baird JK, Tiwari T, Martin GJ, Tamminga CL, Prout TM, Tjaden J, et al. Chloroquine for the treatment of uncomplicated malaria in Guyana. Ann Trop Med Parasitol. 2002;96(4):339-48. Epub 2002/08/13. doi: 10.1179/000349802125001023. PubMed PMID: 12171615.

123. Fontes CJ, Ribeiro LC, Pang LW. Proguanil plus sulfamethoxazole in the treatment of uncomplicated *Plasmodium falciparum* malaria. Southeast Asian J Trop Med Public Health. 2002;33(4):685-8. Epub 2003/05/22. PubMed PMID: 12757209.

124. Fryauff DJ, Leksana B, Masbar S, Wiady I, Sismadi P, Susanti AI, et al. The drug sensitivity and transmission dynamics of human malaria on Nias Island, North Sumatra, Indonesia. Ann Trop Med Parasitol. 2002;96(5):447-62. Epub 2002/08/27. doi: 10.1179/000349802125001249. PubMed PMID: 12194705.

125. Maguire JD, Lacy MD, Sururi, Sismadi P, Krisin, Wiady I, et al. Chloroquine or sulfadoxine-pyrimethamine for the treatment of uncomplicated, *Plasmodium falciparum* malaria during an epidemic in Central Java, Indonesia. Ann

Trop Med Parasitol. 2002;96(7):655-68. Epub 2003/01/23. doi: 10.1179/000349802125002310. PubMed PMID: 12537627.

126. Ezard N, Burns M, Lynch C, Cheng Q, Edstein MD. Efficacy of chloroquine in the treatment of uncomplicated *Plasmodium falciparum* infection in East Timor, 2000. Acta Trop. 2003;88(1):87-90. Epub 2003/08/29. PubMed PMID: 12943982.

127. Mayxay M, Phetsouvanh R, Phompida S, Newton PN, Khanthavong M, Vannachone B, et al. A randomized comparison of oral chloroquine and sulfadoxine-pyrimethamine for the treatment of uncomplicated *Plasmodium falciparum* malaria in Laos. Trans R Soc Trop Med Hyg. 2003;97(3):343-4. Epub 2004/07/02. PubMed PMID: 15228256.

128. Nguyen MH, Davis TM, Cox-Singh J, Hewitt S, Tran QT, Tran BK, et al. Treatment of uncomplicated falciparum malaria in southern Vietnam: can chloroquine or sulfadoxine-pyrimethamine be reintroduced in combination with artesunate? Clin Infect Dis. 2003;37(11):1461-6. Epub 2003/11/14. doi: 10.1086/379323. PubMed PMID: 14614668.

129. Sumawinata IW, Bernadeta, Leksana B, Sutamihardja A, Purnomo, Subianto B, et al. Very high risk of therapeutic failure with chloroquine for uncomplicated *Plasmodium falciparum* and *P. vivax* malaria in Indonesian Papua. Am J Trop Med Hyg. 2003;68(4):416-20. Epub 2003/07/24. PubMed PMID: 12875290.

130. Pukrittayakamee S, Chotivanich K, Chantra A, Clemens R, Looareesuwan S, White NJ. Activities of artesunate and primaquine against asexual- and sexual-stage parasites in falciparum malaria. Antimicrob Agents Chemother. 2004;48(4):1329-34. Epub 2004/03/30. PubMed PMID: 15047537; PubMed Central PMCID: PMCPMC375327.

131. Sutanto I, Supriyanto S, Ruckert P, Purnomo, Maguire JD, Bangs MJ. Comparative efficacy of chloroquine and sulfadoxine-pyrimethamine for uncomplicated *Plasmodium falciparum* malaria and impact on gametocyte carriage rates in the East Nusatenggara province of Indonesia. Am J Trop Med Hyg. 2004;70(5):467-73. Epub 2004/05/25. PubMed PMID: 15155977.

132. Genton B, Baea K, Lorry K, Ginny M, Wines B, Alpers MP. Parasitological and clinical efficacy of standard treatment regimens against *Plasmodium falciparum*, *P. vivax* and *P. malariae* in Papua New Guinea. P N G Med J. 2005;48(3-4):141-50. Epub 2007/01/11. PubMed PMID: 17212060.

133. Marquino W, Ylquimiche L, Hermenegildo Y, Palacios AM, Falconi E, Cabezas C, et al. Efficacy and tolerability of artesunate plus sulfadoxine-pyrimethamine and sulfadoxine-pyrimethamine alone for the treatment of uncomplicated *Plasmodium falciparum* malaria in Peru. Am J Trop Med Hyg. 2005;72(5):568-72. Epub 2005/05/14. PubMed PMID: 15891131.

134. Yeramian P, Meshnick SR, Krudsood S, Chalermrut K, Silachamroon U, Tangpukdee N, et al. Efficacy of DB289 in Thai patients with *Plasmodium vivax* or acute, uncomplicated *Plasmodium falciparum* infections. J Infect Dis. 2005;192(2):319-22. Epub 2005/06/18. doi: 10.1086/430928. PubMed PMID: 15962227.

135. Basano SA, Bianco A, Taylor WR, Olliaro P, Camargo LM. An in vivo test to assess mefloquine 25 mg/kg for the treatment of uncomplicated falciparum malaria in Rondonia, Brazil. Braz J Infect Dis. 2006;10(4):279-82. Epub 2007/02/13. PubMed PMID: 17293912.

136. Lederman ER, Maguire JD, Sumawinata IW, Chand K, Elyazar I, Estiana L, et al. Combined chloroquine, sulfadoxine/pyrimethamine and primaquine against *Plasmodium falciparum* in Central Java, Indonesia. Malar J. 2006;5:108. Epub 2006/11/16. doi: 10.1186/1475-2875-5-108. PubMed PMID: 17105658; PubMed Central PMCID: PMCPMC1665467.

137. Maguire JD, Krisin, Marwoto H, Richie TL, Fryauff DJ, Baird JK. Mefloquine is highly efficacious against chloroquine-resistant *Plasmodium vivax* malaria and *Plasmodium falciparum* malaria in Papua, Indonesia. Clin Infect Dis. 2006;42(8):1067-72. Epub 2006/04/01. doi: 10.1086/501357. PubMed PMID: 16575721.

138. Miller RS, Wongsrichanalai C, Buathong N, McDaniel P, Walsh DS, Knirsch C, et al. Effective treatment of uncomplicated *Plasmodium falciparum* malaria with azithromycin-quinine combinations: a randomized, dose-ranging study. Am J Trop Med Hyg. 2006;74(3):401-6. Epub 2006/03/10. PubMed PMID: 16525097.

139. Thriemer K, Haque R, Wagatsuma Y, Salam MA, Akther S, Attlmayr B, et al. Therapeutic efficacy of quinine plus sulfadoxine-pyremethamine for the treatment of uncomplicated falciparum malaria in Bangladesh. Am J Trop Med Hyg. 2006;75(4):645-9. Epub 2006/10/14. PubMed PMID: 17038687.

140. Ratcliff A, Siswantoro H, Kenangalem E, Wuwung M, Brockman A, Edstein MD, et al. Therapeutic response of multidrug-resistant *Plasmodium falciparum* and *P. vivax* to chloroquine and sulfadoxine-pyrimethamine in southern

Papua, Indonesia. Trans R Soc Trop Med Hyg. 2007;101(4):351-9. Epub 2006/10/10. doi: 10.1016/j.trstmh.2006.06.008. PubMed PMID: 17028048; PubMed Central PMCID: PMCPMC2080856.

141. Karunajeewa HA, Ilett KF, Mueller I, Siba P, Law I, Page-Sharp M, et al. Pharmacokinetics and efficacy of piperaquine and chloroquine in Melanesian children with uncomplicated malaria. Antimicrob Agents Chemother. 2008;52(1):237-43. Epub 2007/10/31. doi: 10.1128/AAC.00555-07. PubMed PMID: 17967917; PubMed Central PMCID: PMCPMC2223898.

142. Tun T, Tint HS, Lin K, Kyaw TT, Myint MK, Khaing W, et al. Efficacy of oral single dose therapy with artemisinin-naphthoquine phosphate in uncomplicated falciparum malaria. Acta Trop. 2009;111(3):275-8. Epub 2009/05/26. doi: 10.1016/j.actatropica.2009.05.007. PubMed PMID: 19464245.

143. Kinzer MH, Chand K, Basri H, Lederman ER, Susanti AI, Elyazar I, et al. Active case detection, treatment of falciparum malaria with combined chloroquine and sulphadoxine/pyrimethamine and vivax malaria with chloroquine and molecular markers of anti-malarial resistance in the Republic of Vanuatu. Malar J. 2010;9:89. Epub 2010/04/08. doi: 10.1186/1475-2875-9-89. PubMed PMID: 20370920; PubMed Central PMCID: PMCPMC2853556.

144. Noedl H, Se Y, Sriwichai S, Schaecher K, Teja-Isavadharm P, Smith B, et al. Artemisinin resistance in Cambodia: a clinical trial designed to address an emerging problem in Southeast Asia. Clin Infect Dis. 2010;51(11):e82-9. Epub 2010/10/30. doi: 10.1086/657120. PubMed PMID: 21028985.

145. Sutanto I, Endawati D, Ling LH, Laihad F, Setiabudy R, Baird JK. Evaluation of chloroquine therapy for vivax and falciparum malaria in southern Sumatra, western Indonesia. Malar J. 2010;9:52. doi: 10.1186/1475-2875-9-52. PubMed PMID: 20152016; PubMed Central PMCID: PMCPMC2831905.

146. Thriemer K, Starzengruber P, Khan WA, Haque R, Marma AS, Ley B, et al. Azithromycin combination therapy for the treatment of uncomplicated falciparum malaria in Bangladesh: an open-label randomized, controlled clinical trial. J Infect Dis. 2010;202(3):392-8. Epub 2010/06/19. doi: 10.1086/653710. PubMed PMID: 20557237.

147. Bethell D, Se Y, Lon C, Tyner S, Saunders D, Sriwichai S, et al. Artesunate dose escalation for the treatment of uncomplicated malaria in a region of reported artemisinin resistance: a randomized clinical trial. PLoS One. 2011;6(5):e19283. Epub 2011/05/24. doi: 10.1371/journal.pone.0019283. PubMed PMID: 21603629; PubMed Central PMCID: PMCPMC3094355.

148. Benjamin J, Moore B, Lee ST, Senn M, Griffin S, Lautu D, et al. Artemisinin-naphthoquine combination therapy for uncomplicated pediatric malaria: a tolerability, safety, and preliminary efficacy study. Antimicrob Agents Chemother. 2012;56(5):2465-71. Epub 2012/02/15. doi: 10.1128/AAC.06248-11. PubMed PMID: 22330921; PubMed Central PMCID: PMCPMC3346652.

149. Mayxay M, Khanthavong M, Chanthongthip O, Imwong M, Lee SJ, Stepniewska K, et al. No evidence for spread of *Plasmodium falciparum* artemisinin resistance to Savannakhet Province, Southern Laos. Am J Trop Med Hyg. 2012;86(3):403-8. Epub 2012/03/10. doi: 10.4269/ajtmh.2012.11-0497. PubMed PMID: 22403308; PubMed Central PMCID: PMCPMC3284353.

150. Mayxay M, Khanthavong M, Chanthongthip O, Imwong M, Pongvongsa T, Hongvanthong B, et al. Efficacy of artemether-lumefantrine, the nationally-recommended artemisinin combination for the treatment of uncomplicated falciparum malaria, in southern Laos. Malar J. 2012;11:184. Epub 2012/06/12. doi: 10.1186/1475-2875-11-184. PubMed PMID: 22681769; PubMed Central PMCID: PMCPMC3523969.

151. Mishra N, Singh JP, Srivastava B, Arora U, Shah NK, Ghosh SK, et al. Monitoring antimalarial drug resistance in India via sentinel sites: outcomes and risk factors for treatment failure, 2009-2010. Bull World Health Organ. 2012;90(12):895-904. Epub 2013/01/04. doi: 10.2471/BLT.12.109124. PubMed PMID: 23284195; PubMed Central PMCID: PMCPMC3524963.

152. Starzengruber P, Swoboda P, Fuehrer HP, Khan WA, Hofecker V, Siedl A, et al. Current status of artemisinin-resistant falciparum malaria in South Asia: a randomized controlled artesunate monotherapy trial in Bangladesh. PLoS One. 2012;7(12):e52236. Epub 2012/12/29. doi: 10.1371/journal.pone.0052236. PubMed PMID: 23272227; PubMed Central PMCID: PMCPMC3525560.

153. Kyaw MP, Nyunt MH, Chit K, Aye MM, Aye KH, Aye MM, et al. Reduced susceptibility of *Plasmodium falciparum* to artesunate in southern Myanmar. PLoS One. 2013;8(3):e57689. Epub 2013/03/23. doi: 10.1371/journal.pone.0057689. PubMed PMID: 23520478; PubMed Central PMCID: PMCPMC3592920.

154. Srivastava P, Ratha J, Shah NK, Mishra N, Anvikar AR, Sharma SK, et al. A clinical and molecular study of artesunate + sulphadoxine-pyrimethamine in three districts of central and eastern India. Malar J. 2013;12:247. Epub

2013/07/20. doi: 10.1186/1475-2875-12-247. PubMed PMID: 23866298; PubMed Central PMCID: PMCPMC3726327.

155. Mishra N, Kaitholia K, Srivastava B, Shah NK, Narayan JP, Dev V, et al. Declining efficacy of artesunate plus sulphadoxine-pyrimethamine in northeastern India. Malar J. 2014;13:284. Epub 2014/07/24. doi: 10.1186/1475-2875-13-284. PubMed PMID: 25052385; PubMed Central PMCID: PMCPMC4127069.

156. Awab GR, Imwong M, Pukrittayakamee S, Alim F, Hanpithakpong W, Tarning J, et al. Clinical trials of artesunate plus sulfadoxine-pyrimethamine for *Plasmodium falciparum* malaria in Afghanistan: maintained efficacy a decade after introduction. Malar J. 2016;15:121. Epub 2016/02/27. doi: 10.1186/s12936-016-1167-z. PubMed PMID: 26917051; PubMed Central PMCID: PMCPMC4766631.

157. Leang R, Canavati SE, Khim N, Vestergaard LS, Borghini Fuhrer I, Kim S, et al. Efficacy and Safety of Pyronaridine-Artesunate for Treatment of Uncomplicated *Plasmodium falciparum* Malaria in Western Cambodia. Antimicrob Agents Chemother. 2016;60(7):3884-90. Epub 2016/03/02. doi: 10.1128/AAC.00039-16. PubMed PMID: 26926629; PubMed Central PMCID: PMCPMC4914696.

158. Mishra N, Srivastava B, Bharti RS, Rana R, Kaitholia K, Anvikar AR, et al. Monitoring the efficacy of antimalarial medicines in India via sentinel sites: Outcomes and risk factors for treatment failure. J Vector Borne Dis. 2016;53(2):168-78. Epub 2016/06/30. PubMed PMID: 27353588.

159. Phong NC, Quang HH, Thanh NX, Trung TN, Dai B, Shanks GD, et al. *In Vivo* Efficacy and Tolerability of Artesunate-Azithromycin for the Treatment of Falciparum Malaria in Vietnam. Am J Trop Med Hyg. 2016;95(1):164-7. Epub 2016/05/25. doi: 10.4269/ajtmh.16-0144. PubMed PMID: 27215294; PubMed Central PMCID: PMCPMC4944683.

160. Rahman R, Martin MJ, Persaud S, Ceron N, Kellman D, Musset L, et al. Continued Sensitivity of *Plasmodium falciparum* to Artemisinin in Guyana, With Absence of Kelch Propeller Domain Mutant Alleles. Open Forum Infect Dis. 2016;3(3):ofw185. Epub 2016/10/06. doi: 10.1093/ofid/ofw185. PubMed PMID: 27704030; PubMed Central PMCID: PMCPMC5047421.

161. Bunnag D, Kanda T, Karbwang J, Thimasarn K, Pungpak S, Harinasuta T. Artemether or artesunate followed by mefloquine as a possible treatment for multidrug resistant falciparum malaria. Trans R Soc Trop Med Hyg. 1996;90(4):415-7. Epub 1996/07/01. PubMed PMID: 8882193.

162. Bunnag D, Kanda T, Karbwang J, Thimasarn K, Pungpak S, Harinasuta T. Two doses of artemether/mefloquine or artesunate/mefloquine combination for multidrug resistant falciparum Malaria. Southeast Asian J Trop Med Public Health. 1997;28(4):727-30. Epub 1998/07/10. PubMed PMID: 9656393.

163. Jiao X, Liu GY, Shan CO, Zhao X, Li XW, Gathmann I, et al. Phase II trial in China of a new, rapidly-acting and effective oral antimalarial, CGP 56697, for the treatment of *Plasmodium falciparum* malaria. Southeast Asian J Trop Med Public Health. 1997;28(3):476-81. Epub 1998/04/30. PubMed PMID: 9561595.

164. Krudsood S, Looareesuwan S, Silachamroon U, Chalermrut K, Pittrow D, Cambon N, et al. Artesunate and mefloquine given simultaneously for three days via a prepacked blister is equally effective and tolerated as a standard sequential treatment of uncomplicated acute *Plasmodium falciparum* malaria: randomized, double-blind study in Thailand. Am J Trop Med Hyg. 2002;67(5):465-72. Epub 2002/12/14. PubMed PMID: 12479545.

165. Wilairatana P, Krudsood S, Chalermrut K, Pengruksa C, Srivilairit S, Silachamroon U, et al. An open randomized clinical trial of Artecom vs artesunate-mefloquine in the treatment of acute uncomplicated falciparum malaria in Thailand. Southeast Asian J Trop Med Public Health. 2002;33(3):519-24. Epub 2003/04/16. PubMed PMID: 12693586.

166. Krudsood S, Chalermrut K, Pengruksa C, Srivilairit S, Silachamroon U, Treeprasertsuk S, et al. Comparative clinical trial of two-fixed combinations dihydroartemisinin-napthoquine-trimethoprim (DNP) and artemether-lumefantrine (Coartem/Riamet) in the treatment of acute uncomplicated falciparum malaria in Thailand. Southeast Asian J Trop Med Public Health. 2003;34(2):316-21. Epub 2003/09/16. PubMed PMID: 12971556; PubMed Central PMCID: PMCPMC3123522.

167. Ashley EA, Krudsood S, Phaiphun L, Srivilairit S, McGready R, Leowattana W, et al. Randomized, controlled dose-optimization studies of dihydroartemisinin-piperaquine for the treatment of uncomplicated multidrug-resistant falciparum malaria in Thailand. J Infect Dis. 2004;190(10):1773-82. doi: 10.1086/425015. PubMed PMID: 15499533.

168. Tangpukdee N, Krudsood S, Thanachartwet V, Pengruksa C, Phophak N, Kano S, et al. Efficacy of Artequick versus artesunate-mefloquine in the treatment of acute uncomplicated falciparum malaria in Thailand. Southeast Asian J Trop Med Public Health. 2008;39(1):1-8. Epub 2008/06/24. PubMed PMID: 18567436; PubMed Central PMCID: PMCPMC3129605.

169. Valecha N, Krudsood S, Tangpukdee N, Mohanty S, Sharma SK, Tyagi PK, et al. Arterolane maleate plus piperaquine phosphate for treatment of uncomplicated *Plasmodium falciparum* malaria: a comparative, multicenter, randomized clinical trial. Clin Infect Dis. 2012;55(5):663-71. Epub 2012/05/16. doi: 10.1093/cid/cis475. PubMed PMID: 22586253.

170. Benjamin JM, Moore BR, Salman S, Page-Sharp M, Tawat S, Yadi G, et al. Population pharmacokinetics, tolerability, and safety of dihydroartemisinin-piperaquine and sulfadoxine-pyrimethamine-piperaquine in pregnant and nonpregnant Papua New Guinean women. Antimicrob Agents Chemother. 2015;59(7):4260-71. Epub 2015/05/13. doi: 10.1128/AAC.00326-15. PubMed PMID: 25963981; PubMed Central PMCID: PMCPMC4468729.

171. Thimasarn K, Sirichaisinthop J, Chanyakhun P, Palananth C, Rooney W. A comparative study of artesunate and artemether in combination with mefloquine on multidrug resistant falciparum malaria in eastern Thailand. Southeast Asian J Trop Med Public Health. 1997;28(3):465-71. Epub 1998/04/30. PubMed PMID: 9561593.

172. Karbwang J, Na-Bangchang K, Thanavibul A, Mull R, Gathmann I. Dose-finding study of the efficacy of fixed-combination artemether/lumefantrine for the treatment of multidrug-resistant *Plasmodium falciparum* malaria in Thailand. Clin Drug Inv. 2000;19(5):343-8.

173. Kshirsagar NA, Gogtay NJ, Moorthy NS, Garg MR, Dalvi SS, Chogle AR, et al. A randomized, double-blind, parallel-group, comparative safety, and efficacy trial of oral co-artemether versus oral chloroquine in the treatment of acute uncomplicated *Plasmodium falciparum* malaria in adults in India. Am J Trop Med Hyg. 2000;62(3):402-8. Epub 2000/10/19. PubMed PMID: 11037786.

174. Denis MB, Davis TM, Hewitt S, Incardona S, Nimol K, Fandeur T, et al. Efficacy and safety of dihydroartemisinin-piperaquine (Artekin) in Cambodian children and adults with uncomplicated falciparum malaria. Clin Infect Dis. 2002;35(12):1469-76. Epub 2002/12/10. doi: 10.1086/344647. PubMed PMID: 12471565.

175. Stohrer JM, Dittrich S, Thongpaseuth V, Vanisaveth V, Phetsouvanh R, Phompida S, et al. Therapeutic efficacy of artemether-lumefantrine and artesunate-mefloquine for treatment of uncomplicated *Plasmodium falciparum* malaria in Luang Namtha Province, Lao People's Democratic Republic. Trop Med Int Health. 2004;9(11):1175-83. Epub 2004/11/19. doi: 10.1111/j.1365-3156.2004.01320.x. PubMed PMID: 15548313.

176. Hutagalung R, Paiphun L, Ashley EA, McGready R, Brockman A, Thwai KL, et al. A randomized trial of artemether-lumefantrine versus mefloquine-artesunate for the treatment of uncomplicated multi-drug resistant *Plasmodium falciparum* on the western border of Thailand. Malar J. 2005;4:46. Epub 2005/09/24. doi: 10.1186/1475-2875-4-46. PubMed PMID: 16179089; PubMed Central PMCID: PMCPMC1261533.

177. Denis MB, Tsuyuoka R, Poravuth Y, Narann TS, Seila S, Lim C, et al. Surveillance of the efficacy of artesunate and mefloquine combination for the treatment of uncomplicated falciparum malaria in Cambodia. Trop Med Int Health. 2006;11(9):1360-6. Epub 2006/08/26. doi: 10.1111/j.1365-3156.2006.01690.x. PubMed PMID: 16930257.

178. Denis MB, Tsuyuoka R, Lim P, Lindegardh N, Yi P, Top SN, et al. Efficacy of artemether-lumefantrine for the treatment of uncomplicated falciparum malaria in northwest Cambodia. Trop Med Int Health. 2006;11(12):1800-7. Epub 2006/12/21. doi: 10.1111/j.1365-3156.2006.01739.x. PubMed PMID: 17176344.

179. Thapa S, Hollander J, Linehan M, Cox-Singh J, Bista MB, Thakur GD, et al. Comparison of artemether-lumefantrine with sulfadoxine-pyrimethamine for the treatment of uncomplicated falciparum malaria in eastern Nepal. Am J Trop Med Hyg. 2007;77(3):423-30. Epub 2007/09/11. PubMed PMID: 17827354.

180. Rogers WO, Sem R, Tero T, Chim P, Lim P, Muth S, et al. Failure of artesunate-mefloquine combination therapy for uncomplicated *Plasmodium falciparum* malaria in southern Cambodia. Malar J. 2009;8:10. Epub 2009/01/14. doi: 10.1186/1475-2875-8-10. PubMed PMID: 19138388; PubMed Central PMCID: PMCPMC2628668.

181. Assefa A, Kassa M, Tadese G, Mohamed H, Animut A, Mengesha T. Therapeutic efficacy of Artemether/Lumefantrine (Coartem(R)) against *Plasmodium falciparum* in Kersa, South West Ethiopia. Parasit Vectors. 2010;3(1):1. Epub 2010/01/07. doi: 10.1186/1756-3305-3-1. PubMed PMID: 20051120; PubMed Central PMCID: PMCPMC2881066.

182. Na-Bangchang K, Ruengweerayut R, Mahamad P, Ruengweerayut K, Chaijaroenkul W. Declining in efficacy of a three-day combination regimen of mefloquine-artesunate in a multi-drug resistance area along the Thai-Myanmar

border. Malar J. 2010;9:273. Epub 2010/10/12. doi: 10.1186/1475-2875-9-273. PubMed PMID: 20929590; PubMed Central PMCID: PMCPMC2959072.

183. Eshetu T, Abdo N, Bedru KH, Fekadu S, Wieser A, Pritsch M, et al. Open-label trial with artemether-lumefantrine against uncomplicated *Plasmodium falciparum* malaria three years after its broad introduction in Jimma Zone, Ethiopia. Malar J. 2012;11:240. Epub 2012/07/25. doi: 10.1186/1475-2875-11-240. PubMed PMID: 22824059; PubMed Central PMCID: PMCPMC3438107.

184. Leang R, Barrette A, Bouth DM, Menard D, Abdur R, Duong S, et al. Efficacy of dihydroartemisinin-piperaquine for treatment of uncomplicated *Plasmodium falciparum* and *Plasmodium vivax* in Cambodia, 2008 to 2010. Antimicrob Agents Chemother. 2013;57(2):818-26. Epub 2012/12/05. doi: 10.1128/AAC.00686-12. PubMed PMID: 23208711; PubMed Central PMCID: PMCPMC3553743.

185. Valecha N, Srivastava B, Dubhashi NG, Rao BH, Kumar A, Ghosh SK, et al. Safety, efficacy and population pharmacokinetics of fixed-dose combination of artesunate-mefloquine in the treatment of acute uncomplicated *Plasmodium falciparum* malaria in India. J Vector Borne Dis. 2013;50(4):258-64. Epub 2014/02/07. PubMed PMID: 24499847.

186. Lon C, Manning JE, Vanachayangkul P, So M, Sea D, Se Y, et al. Efficacy of two versus three-day regimens of dihydroartemisinin-piperaquine for uncomplicated malaria in military personnel in northern Cambodia: an open-label randomized trial. PLoS One. 2014;9(3):e93138. Epub 2014/03/29. doi: 10.1371/journal.pone.0093138. PubMed PMID: 24667662; PubMed Central PMCID: PMCPMC3965521.

187. Saunders DL, Vanachayangkul P, Lon C, Program USAMMR, National Center for Parasitology E, Malaria C, et al. Dihydroartemisinin-piperaquine failure in Cambodia. N Engl J Med. 2014;371(5):484-5. Epub 2014/07/31. doi: 10.1056/NEJMc1403007. PubMed PMID: 25075853.

188. Ebstie YA, Zeynudin A, Belachew T, Desalegn Z, Suleman S. Assessment of therapeutic efficacy and safety of artemether-lumefantrine (Coartem(R)) in the treatment of uncomplicated *Plasmodium falciparum* malaria patients in Bahir Dar district, Northwest Ethiopia: an observational cohort study. Malar J. 2015;14:236. Epub 2015/06/06. doi: 10.1186/s12936-015-0744-x. PubMed PMID: 26045199; PubMed Central PMCID: PMCPMC4464854.

189. Mekonnen SK, Medhin G, Berhe N, Clouse RM, Aseffa A. Efficacy of artemether-lumefantrine therapy for the treatment of uncomplicated *Plasmodium falciparum* malaria in Southwestern Ethiopia. Malar J. 2015;14:317. Epub 2015/08/15. doi: 10.1186/s12936-015-0826-9. PubMed PMID: 26271736; PubMed Central PMCID: PMCPMC4536736.

190. Wudneh F, Assefa A, Nega D, Mohammed H, Solomon H, Kebede T, et al. Open-label trial on efficacy of artemether/lumefantrine against the uncomplicated *Plasmodium falciparum* malaria in Metema district, Northwestern Ethiopia. Ther Clin Risk Manag. 2016;12:1293-300. Epub 2016/09/08. doi: 10.2147/TCRM.S113603. PubMed PMID: 27601913; PubMed Central PMCID: PMCPMC5005000.

191. Teklemariam M, Assefa A, Kassa M, Mohammed H, Mamo H. Therapeutic efficacy of artemether-lumefantrine against uncomplicated *Plasmodium falciparum* malaria in a high-transmission area in northwest Ethiopia. PLoS One. 2017;12(4):e0176004. Epub 2017/04/27. doi: 10.1371/journal.pone.0176004. PubMed PMID: 28445503; PubMed Central PMCID: PMCPMC5405980.

192. Itoh M, Negreiros do Valle S, Farias S, Holanda de Souza TM, Rachid Viana GM, Lucchi N, et al. Efficacy of Artemether-Lumefantrine for Uncomplicated *Plasmodium falciparum* Malaria in Cruzeiro do Sul, Brazil, 2016. Am J Trop Med Hyg. 2018;98(1):88-94. Epub 2017/11/17. doi: 10.4269/ajtmh.17-0623. PubMed PMID: 29141762; PubMed Central PMCID: PMCPMC5928740.

193. Wilairatana P, Krudsood S, Chokejindachai W, Bussaratid V, Silachamroon U, Viriyavejakul P, et al. A clinical trial of combination of artesunate and mefloquine in the treatment of acute uncomplicated falciparum malaria: a short and practical regimen. Southeast Asian J Trop Med Public Health. 1998;29(4):696-701. Epub 2000/04/20. PubMed PMID: 10772548.

194. Lefevre G, Looareesuwan S, Treeprasertsuk S, Krudsood S, Silachamroon U, Gathmann I, et al. A *clinical and pharmacokinetic trial of six doses of artemether-lumefantrine for multidrug-resistant Plasmodium* falciparum malaria in Thailand. Am J Trop Med Hyg. 2001;64(5-6):247-56. Epub 2001/07/21. PubMed PMID: 11463111.

195. Durrani N, Leslie T, Rahim S, Graham K, Ahmad F, Rowland M. Efficacy of combination therapy with artesunate plus amodiaquine compared to monotherapy with chloroquine, amodiaquine or sulfadoxine-pyrimethamine for treatment of uncomplicated *Plasmodium falciparum* in Afghanistan. Trop Med Int Health. 2005;10(6):521-9. Epub 2005/06/09. doi: 10.1111/j.1365-3156.2005.01429.x. PubMed PMID: 15941414.
