# Supplementary material for: The risk of Plasmodium vivax parasitaemia after P. falciparum malaria: An individual patient data meta-analysis from the WorldWide Antimalarial Resistance Network
Source: PLoS Med. 2020 Nov 19;17(11):e1003393. doi: 10.1371/journal.pmed.1003393 (PMC7676739; doi:10.1371/journal.pmed.1003393)
Supplement: S2 Table — (PDF) [file pmed.1003393.s010.pdf]

S2 Table. Studies included in meta-analysis

Author - Year	Country	Years enrolled	Randomised	Blinded	Ages enrolled (years)			Mixed infections	Follow up (days)	Patients enrolled*	Patients included	Treatment arms	ACT included	Sites
					<5	5-15	>15							
Nosten - 1994[45]	*Thailand*	*1992-1993*	*Yes*	*NS*	*Yes*	*Yes*	*Yes*	*NS*	*28/63†*	*652*	*178*	*3*	*AM (MQ25 d0 + AS10 d0/1/2)*	*1*
Luxemburger - 1994[46]	*Thailand*	*1991*	*Yes*	*NS*	*Yes*	*Yes*	*Yes*	*NS*	*28*	*552*	*269*	*2*	*AM (MQ25 d1 + AS12 d0/1/2)*	*2*
Price - 1995[47]	*Thailand*	*1993-1994*	*Yes*	*NS*	*Yes*	*Yes*	*Yes*	*NS*	*63*	*550*	*181*	*3*	*AM (MQ25 d2 + AS12 d0/1/2)*	*1*
Price - 1997[48]	*Thailand*	*1992-1995*	*No*	*No*	*Yes*	*Yes*	*Yes*	*NS*	*63*	*1967*	*1532*	*1*	*AM (MQ25 d2 + AS12 d0/1/2; MQ15/10 d2/3 + AS12 d0/1/2)*	*1*
van Vugt - 1998[19]	Thailand	1995-1996	Yes	No	Yes	Yes	Yes	No	63	617	606	2	AL, AM (MQ15/10 d2/3 + AS12 d0/1/2)	1
van Vugt - 1999[21]	Thailand	1996-1997	Yes	Yes	Yes	Yes	Yes	No	28/63†	359	258	3	AL	2
van Vugt - 2000[49]	*Thailand*	*1997-1998*	*Yes*	*No*	*Yes*	*Yes*	*Yes*	*NS*	*28*	*200*	*199*	*2*	*AL, AM (MQ15/10 d2/3 + AS12 d0/1/2)*	*2*
van Vugt - 2002[20]	Thailand	1998-2000	Yes	No	Yes	Yes	Yes	No	42	1596	532	3	AM (MQ15/10 d1/2 + AS12 d0/1/2)	1
Suputtamongkol - 2003[22]	Thailand	1999-2001	Yes	No	No	Yes	Yes	No	42	556	320	2	AM (MQ15/10 + AS12 d0/1/2)	7
Mayxay - 2004[23]	Laos	2002-2003	Yes	No	Yes	Yes	Yes	No	42	330	220	3	AL, AM (MQ15/10 d1/2 + AS12 d0/1/2)	1
Smithuis - 2004[24]	Myanmar	2000-2001	Yes	Yes	Yes	Yes	Yes	Yes	42	803	803	4	AM (MQ15 d0 + AS4 d0; MQ25 d0 + AS4 d0; MQ15/10 d1/2 + AS12 d0/1/2 (partial sup); MQ15/10 d1/2 + AS12 d0/1/2 (full sup))	2
Smithuis – 2004b[36]	Myanmar	1998	Yes	No	Yes	Yes	Yes	No	42	316	78	4	AM (MQ15 d0 + AS4 d0)	1
Ashley - 2005[25]	Thailand	2003-2004	Yes	No	Yes	Yes	Yes	Yes	63	499	497	3	AM (MQ8/8/8 d0/1/2 + AS12 d0/1/2), DP	4
van den Broek – 2005[44]	Thailand	2003	Yes	No	Yes	Yes	Yes	No	42	364	242	3	AL, AM (MQ15/10 d0/1 + AS12 d0/1/2)	2
Price - 2006[50]	*Thailand*	*1995-2002*	*NS*	*NS*	*Yes*	*Yes*	*Yes*	*NS*	*42*	*1588*	*892*	*3*	*AL*	*1*
Mayxay - 2006[26]	Laos	2004	Yes	No	Yes	Yes	Yes	No	42	220	220	2	DP, AM (MQ15/10 d1/2 + AS12 d0/1/2)	1
Ashley - 2006[51]	*Thailand*	*2004-2005*	*Yes*	*No*	*Yes*	*Yes*	*Yes*	*Yes*	*63*	*500*	*492*	*2*	*AM (MQ8/8/8 d0/1/2 + AS12 d0/1/2; MQ15/10 d2/3 + AS12 d0/1/2)*	*6*
Smithuis – 2006[37]	Myanmar	2003-2004	Yes	No	Yes	Yes	Yes	Yes	42	652	652	4	AM (MQ25 d0 + AS12 d0/1/2 (full sup); MQ25 d0 + AS12 d0/1/2 (partial sup)), DP	1
Grande - 2007[27]	Peru	2003-2005	Yes	No	No	Yes	Yes	No	63	522	522	2	DP, AM (MQ8/8/8 d0/1/2 + AS12 d0/1/2)	1
Haque – 2007[38]	Bangladesh	2005	No	No	No	No	Yes	No	42	67	67	1	AL	2

Janssens - 2007[28]	Cambodia	2002-2003	Yes	No	Yes	Yes	Yes	Yes	63	464	454	2	DP, AM (MQ12.5/12.5 d0/0 + AS12 d0/0/1/2)	2
Ratcliff - 2007[52]	*Indonesia*	*2004-2005*	*Yes*	*No*	*Yes*	*Yes*	*Yes*	*Yes*	*42*	*774*	*575*	*2*	*DP, AL*	*1*
Hasugian - 2007[53]	*Indonesia*	*2005*	*Yes*	*No*	*Yes*	*Yes*	*Yes*	*Yes*	*42*	*340*	*214*	*2*	*DP, AA*	*2*
Karunajeewa - 2008[30]	Papua New Guinea	2005-2007	Yes	No	Yes	No	No	Yes	42	742	269	4	DP, AL	2
Dondorp - 2009[29]	Multicentred	2007-2008	Yes	No	No	Yes	Yes	No	63	80	20	2	AM (MQ15/10 d3/4 + AS12 d0/1/2)	2
Thanh - 2009[54]	*Vietnam*	*2006-2007*	*Yes*	*No*	*No*	*Yes*	*Yes*	*No*	*42*	*116*	*116*	*2*	*DP, AA*	*1*
Carrara-2009[55]	*Thailand*	*1995-2007*	*NS*	*NS*	*Yes*	*Yes*	*Yes*	*Yes*	*42*	*3264*	*485*	*1*	*AM (MQ25 d0 + AS12 d0/1/2; MQ15/10 d1/2 + AS12 d0/1/2; MQ8/8/8 d0/1/2 + AS12 d0/1/2)*	*NS*
Mayxay – 2010[39]	Laos	2005-2006	Yes	No	Yes	Yes	Yes	Yes	63	300	300	2	AM (MQ15/10 d1/2 + AS12 d0/1/2), DP	1
Smithuis - 2010[32]	Myanmar	2008-2009	Yes	No	Yes	Yes	Yes	Yes	63	808	808	10	DP, AL, AM (MQ25 d0 + AS12 d0/1/2; MQ8.8/8.8/8.8 d0/1/2 + AS12 d0/1/2), AA	3
Hwang - 2011[31]	Ethiopia	2009	No	No	Yes	Yes	Yes	No	42	120	120	1	AL	2
Salman - 2011[56]	*Papua New Guinea*	*NS*	*No*	*No*	*No*	*Yes*	*No*	*Yes*	*42*	*13*	*11*	*1*	*AL*	*1*
Hien - 2012[33]	Vietnam	2010-2011	Yes	No	No	Yes	Yes	No	42	166	55	3	DP	1
Anvikar - 2012[57]	*India*	*2007-2008*	*Yes*	*No*	*Yes*	*Yes*	*Yes*	*No*	*28*	*300*	*202*	*2*	*AA*	*2*
Thanh - 2012[40]	Vietnam	2008-2009	Yes	No	No	Yes	Yes	No	42	128	65	2	AA	1
Laman - 2014[34]	Papua New Guinea	2011-2013	Yes	No	Yes	No	No	Yes	42	267	97	2	AL	1
Jullien - 2014[58]	*India*	*2007-2008*	*No*	*No*	*No*	*No*	*Yes*	*NS*	*42*	*77*	*77*	*1*	*AM (MQ8/8/8 d0/1/2 + AS12 d0/1/2)*	*2*
Spring - 2015[41]#	Cambodia	2012-2014	No	No	No	No	Yes	Yes	42	107	107	2	DP	1
Amaratunga - 2016[42]	Cambodia	2012-2013	No	No	Yes	Yes	Yes	No	63	241	241	1	DP	3
Ladeia-Andrade - 2016[43]	Brazil	2010-2013	No	No	Yes	Yes	Yes	No	42	162	162	1	AM (MQ8/8/8 d0/1/2 + AS12 d0/1/2)	1
Poespoprodjo - 2018[35]	Indonesia	2015-2016	No	No	Yes	Yes	Yes	No	42	129	61	1	DP	1
HCGRD‡	*Indonesia*	*2011-2012*	*NS*	*NS*	*Yes*	*Yes*	*Yes*	*NS*	*42*	*117*	*70*	*NS*	*AA*	*NS*
ZMNBX‡	*Vietnam*	*2002-2003*	*Yes*	*No*	*Yes*	*Yes*	*Yes*	*NS*	*56*	*2951*	*2072*	*3*	*DP, AM (MQ15/10 + AS12 d0/1/2)*	*1*

AA – artesunate-amodiaquine; AL – artemether-lumefantrine; AM – artesunate-mefloquine; AS12 – artesunate total dose 12 mg/kg; d0/1 – dosed on day 0 and day 1; DP – dihydroartemisinin-piperaquine; MQ15/10 – mefloquine split into 15 mg/kg and 10mg/kg doses; NS – not stated; * Includes patients with *P. falciparum* mono-infection, *P. vivax* mono-infection and any mixed infection with *P. falciparum*; † Some participants followed for 28 days and others followed for 63 days; # Single low dose primaquine given to 50 patients; ‡ Unpublished study; Italicised studies were not included in original systematic review.
