# Supplementary material for: The risk of Plasmodium vivax parasitaemia after P. falciparum malaria: An individual patient data meta-analysis from the WorldWide Antimalarial Resistance Network
Source: PLoS Med. 2020 Nov 19;17(11):e1003393. doi: 10.1371/journal.pmed.1003393 (PMC7676739; doi:10.1371/journal.pmed.1003393)
Supplement: S3 Table — (PDF) [file pmed.1003393.s011.pdf]

S3 Table. Study sites included in analysis

Author-Year	Country	Study site	Latitude	Longitude	Year Start	Year End	Supervision	Pf incidence (per 1000 person years)*	Pv incidence (per 1000 person years)*	Region of relapse periodicity†	Final category of relapse periodicity‡
Amaratunga - 2016[42]	Cambodia	Pursat	12.5333	103.9167	2012	2013	Full	17.210	1.908	10	High
Amaratunga – 2016[42]	Cambodia	Preah Vihear	14.0086	104.8455	2012	2013	Full	8.939	40.436	10	High
Amaratunga - 2016[42]	Cambodia	Ratanakiri	13.7333	107.0000	2012	2013	Full	9.333	2.358	10	High
Anvikar-2012[57]	India	Ranchi	23.3511	85.3205	2007	2008	Not stated	72.306	90.769	8	Low
Anvikar-2012[57]	India	Rourkela	22.2494	84.8830	2007	2008	Not stated	29.590	26.027	8	Low
Ashley-2005[25]	Thailand	Thai-Myanmar border	17.1250	98.3936	2003	2004	Full	0.084	0.205	10	High
Ashley-2006[51]	Thailand	Thai-Myanmar border	17.1250	98.3936	2004	2005	Full	0.079	0.213	10	High
Carrara-2009[55]	Thailand	Thai-Myanmar border	17.1250	98.3936	1995	2007	Not stated	0.546	3.398	10	High
Dondorp-2009[29]	Cambodia	Pailin	12.8467	102.6130	2007	2008	Full	54.697	7.791	10	High
Dondorp-2009[29]	Thailand	Wang Pha	16.7752	98.7041	2007	2008	Full	3.779	7.812	10	High
Grande-2007[27]	Peru	Iquitos	-3.7500	-73.2500	2003	2005	Full	41.580	68.398	3	Low
Haque-2007[38]	Bangladesh	Bandarban	22.2000	92.2167	2005	2005	Not stated	24.255	6.175	10	High
Hasugian-2007[53]	Indonesia	Timika	-4.6140	136.8515	2005	2005	Full	18.060	28.112	12	High
HCGRD	Indonesia	East Nusa Tenggara	-8.7439	121.1133	2011	2012	Not stated	0.923	43.086	10	High
Hien-2012[33]	Vietnam	Phuoc Long	11.8338	106.9921	2010	2011	Full	3.933	1.213	10	High
Hwang-2011[31]	Ethiopia	Bishoftu	9.0999	37.2567	2009	2009	Partial	8.745	8.1189	7	Low
Hwang-2011[31]	Ethiopia	Bulbula	7.7226	38.6539	2009	2009	Partial	8.745	8.119	7	Low
Janssens-2007[28]	Cambodia	Oddor Meanchey	14.2333	104.0833	2002	2003	Full	39.736	13.571	10	High
Janssens-2007[28]	Cambodia	Siem Reap	13.3622	103.8597	2002	2003	Full	6.419	2.369	10	High
Jullien-2014[58]	India	Goa	15.4254	73.9830	2007	2008	Not stated	23.876	9.780	8	Low
Jullien-2014[58]	India	New Delhi	28.6357	77.2246	2007	2008	Not stated	0.213	0.302	8	Low
Karunajeewa-2008[30]	Papua New Guinea	East Sepik	-4.4560	143.0090	2005	2007	Partial	123.498	151.427	12	High
Karunajeewa-2008[30]	Papua New Guinea	Madang	-5.2341	145.7888	2005	2007	Partial	123.498	151.427	12	High
Ladeia-Andrade-2016[43]	Brazil	Cruzeiro do Sul	-7.6280	-72.6761	2010	2013	Full	12.750	82.666	3	Low
Laman-2014[34]	Papua New Guinea	Madang Province	-5.2341	145.7888	2011	2013	Partial	104.573	85.293	12	High
Luxemburger-1994[46]	Thailand	Shoklo	17.2048	98.2613	1991	1991	Full	0.546	3.398	10	High
Mayxay-2004[23]	Laos	Savannakhet Province	16.5065	105.5943	2002	2003	Full	30.158	1.600	10	High
Mayxay-2006[26]	Laos	Savannakhet Province	16.5065	105.5943	2004	2004	Full	25.230	1.271	10	High
Mayxay-2010[39]	Laos	Xepon	16.6961	106.2082	2005	2006	Full	19.484	0.289	10	High

Nosten-1994[45]	Thailand	Thai-Myanmar border	17.2048	98.2613	1992	1993	Full	0.546	3.398	10	High
Poespoprodjo-2018[35]	Indonesia	Timika	-4.6140	136.8515	2015	2016	Full	135.234	83.297	12	High
Price-1995[47]	Thailand	Thai-Myanmar border	17.2048	98.2613	1993	1994	Full	0.546	3.398	10	High
Price-1997[48]	Thailand	Thai-Myanmar border	17.2048	98.2613	1992	1995	Full	0.546	3.398	10	High
Price-2006[50]	Thailand	Thai-Myanmar border	17.1250	98.3936	1995	2002	Full	0.546	3.398	10	High
Ratcliff-2007[52]	Indonesia	Timika	-4.6140	136.8515	2004	2005	Partial	18.060	28.112	12	High
Salman-2011[56]	Papua New Guinea	Madang Province	-5.2341	145.7888	NS	NS	Full	153.797	145.683	12	High
Smithuis-2004[24]	Myanmar	Sittwe in Rakhine State	20.1103	93.2584	2000	2001	Partial	40.909	25.869	10	High
Smithuis-2004b[36]	Myanmar	Kachin state	26.0900	97.3261	1998	1998	Full	23.534	10.125	10	High
Smithuis-2006[37]	Myanmar	Rakhine state	19.8101	93.9878	2003	2004	Partial	28.840	21.314	10	High
Smithuis-2010[32]	Myanmar	Kachin state	26.0900	97.3261	2008	2009	Partial	28.398	4.860	10	High
Smithuis-2010[32]	Myanmar	Rakhine state	19.8101	93.9878	2008	2009	Partial	89.753	53.454	10	High
Smithuis-2010[32]	Myanmar	Shan state	21.7347	98.6895	2008	2009	Partial	22.977	1.613	10	High
Spring-2015[41]	Cambodia	Oddar Meanchey	14.1667	103.5000	2012	2014	Full	5.561	2.184	10	High
Suputtamongkol-2003[22]	Thailand	Saiyok District	14.0701	99.4869	1999	2001	Not stated	1.005	1.772	10	High
Thanh-2009[54]	Vietnam	Phuoc Chien Commune	11.6994	108.9027	2006	2007	Full	1.087	0.487	10	High
Thanh-2012[40]	Vietnam	Phuoc Chien	11.8175	109.0406	2008	2009	Full	1.591	0.364	10	High
van den Broek-2005[44]	Bangladesh	Dighinala	23.240847	92.064072	2003	2003	Full	35.919	10.880	10	High
van den Broek-2005[44]	Bangladesh	Panchari	23.70731	90.41548	2003	2003	Full	35.919	10.880	10	High
van Vugt-1998[19]	Thailand	Thai-Myanmar border	17.1250	98.3833	1995	1996	Full	0.546	3.398	10	High
van Vugt-1999[21]#	Thailand	Bangkok	13.7234	100.4762	1996	1997	Full	1.766	1.261	10	High
van Vugt-1999[21]	Thailand	Thai-Myanmar border	17.1250	98.3833	1996	1997	Full	0.546	3.398	10	High
van Vugt-2000[49]#	Thailand	Bangkok	13.7234	100.4762	1997	1998	Full	1.766	1.261	10	High
van Vugt-2000[49]	Thailand	Thai-Myanmar border	17.1250	98.3833	1997	1998	Full	0.546	3.398	10	High
van Vugt-2002[20]	Thailand	Thai-Myanmar border	16.7752	98.7041	1998	2000	Full	0.546	3.398	10	High
ZMNBX	Vietnam	Dac O	12.0461	107.0935	2002	2003	Not stated	2.254	0.787	10	High

* Subnational *P. falciparum* and *P. vivax* incidence rates estimated by Malaria Atlas Project (MAP); † Regional relapse periodicity estimated from Battle et al, 2014[14]; ‡ Short relapse periodicity ≤47 days; # Due to low malaria endemicity in Bangkok city, incidence rates based on national data.
