# Supplementary material for: The risk of Plasmodium vivax parasitaemia after P. falciparum malaria: An individual patient data meta-analysis from the WorldWide Antimalarial Resistance Network
Source: PLoS Med. 2020 Nov 19;17(11):e1003393. doi: 10.1371/journal.pmed.1003393 (PMC7676739; doi:10.1371/journal.pmed.1003393)
Supplement: S4 Table — (PDF) [file pmed.1003393.s012.pdf]

S4 Table. Studies targeted for analysis but not available

First Author	Sites	Region	Country	Follow up (days)	Randomised	Recruitment period	ACT treatment arms	*Pf* patients enrolled	Patients eligible	Male (%)	Age enrolled (years)		Day 28 recurrence % (n/N)	Day 42 recurrence % (n/N)	Day 63 recurrence % (n/N)	Reason not included
											Mean (SD)	Median (range)				
Thimasarn – 1997[171]	2	Asia-Pacific	Thailand	28	Yes	1993-1994	AM	382	193	NS	30.2 (-)	NS	1.1 (2/175)			Data not available
Wilairatana – 1998[193]	1	Asia-Pacific	Thailand	28	No	1997-1998	AM	150	150	65.3	26.6 (10.2)	NS	6.9 (9/130)			Data not available
Karbwang – 2000[172]	2	Asia-Pacific	Thailand	28	Yes	NS	AL	260	260	NS	NS	22 (14-60)	12.7 (30/236)			Data not available
Kshirsagar – 2000[173]	1	Asia-Pacific	India	29	Yes	1996-1997	AL	179	89	95.0	NS	- (16-70)	0.7 (1/142)			Data not available
Lefevre – 2001[194]	1	Asia-Pacific	Thailand	28	Yes	1998-1999	AL, AM	219	219	71.2	24.7 (-)	- (12-71)	2.9 (6/208)			Essential data not available
Denis – 2002[174]	2	Asia-Pacific	Cambodia	28	No	2001-2002	DP	106	106	62.3	13.9 (-)	NS	1.0 (1/97)			Data not available
Stohrer – 2004[175]	1	Asia-Pacific	India	42	Yes	2003	AL, AM	108	108	48.1	NS	9.5 (2-66)		2.9 (3/103)		Data not available
Durrani – 2005[195]	1	Asia-Pacific	Afghanistan	42	Yes	2002-2003	AA	268	79	62.0	14.7 (1.5)	NS		3.0 (2/67)		Essential data not available
Hutagalung – 2005[176]	1	Asia-Pacific	Thailand	42	Yes	2001-2002	AL, AM	490	490	68.6	23.4 (-)	- (2-72)		26.3 (119/452)		Data not available
Denis – 2006a[177]	1	Asia-Pacific	Cambodia	28	No	2001-2004	AM	1025	1025	NS	NS	NS		0.1 (1/977)		Data not available
Denis – 2006b[178]	1	Asia-Pacific	Cambodia	28	Yes	2003-2004	AL, AM	190	190	74.7	21.4 (-)	NS		1.7 (3/178)		Data not available
Thapa – 2007[179]	1	Asia-Pacific	Nepal	28	Yes	2005	AL	99	66	53.0	26.5 (13.8)	NS	0.0 (0/66)			Data not available
Rogers – 2009[180]	1	Asia-Pacific	Cambodia	42	No	2006-2008	AM	150	150	93.4	27.1 (9.5)	NS		5.6 (8/143)		Data not available
Assefa – 2010[181]	1	Africa	Ethiopia	28	No	2007-2008	AL	90	90	46.7	9.0 (-)	- (1-30)	5.9 (5/85)			Data not available
Na Bangchang - 2010[182]	1	Asia-Pacific	Thailand	42	No	2008-2009	AM	150	150	56.7	NS	25 (16-50)		3.8 (5/132)		Data not available
Eshetu - 2012[183]	4	Africa	Ethiopia	42	No	2008-2009	AL	348	348	59.8	17.1 (-)	NS	0.0 (0/317)	0.0 (0/316)		Data not available
Leang - 2013[184]	4	Asia-Pacific	Cambodia	42	No	2008-2011	DP	438	438	68.0	23.4 (-)	- (2-60)		1.4 (6/426)		Data not available
Valecha - 2013[185]	2	Asia-Pacific	India	63	No	2007-2008	AM	77	77	96.1	28.2 (8.8)	NS			10.6 (7/66)	Data not available
Lon - 2014[186]	1	Asia-Pacific	Cambodia	42	Yes	2010-2011	DP	20	20	NS	NS	NS		5.0 (1/20)		Data not available
Saunders - 2014[187]	1	Asia-Pacific	Cambodia	42	Yes	2013	DP	50	50	NS	NS	NS		8.7 (4/46)		Data not available
Ebstie - 2015[188]	1	Africa	Ethiopia	28	No	2012	AL	134	134	60.0	NS	NS	0.8 (1/131)			Data not available
Mekonnen - 2015[189]	1	Africa	Ethiopia	28	No	2011	AL	93	93	59.8	17.3 (-)	- (1-60)	1.1 (1/89)			Data not available
Wudneh - 2016[190]	1	Africa	Ethiopia	28	No	2014-2015	AL	91	91	82.4	13.0 (-)	- (2-24)	1.2 (1/82)			Data not available
Teklemariam - 2017[191]	1	Africa	Ethiopia	28	No	2014-2015	AL	92	92	62.0	15.1 (-)	- (2-28)	2.5 (2/81)			Data not available
Itoh - 2018[192]	1	The Americas	Brazil	28	No	2015-2016	AL	85	85	49.4	NS	30 (5-79)	1.3 (1/75)			Data not available

AA – artesunate-amodiaquine; ACT – artemisinin-based combination therapy; AL – artemether-lumefantrine; AM – artesunate-mefloquine; DP – dihydroartemisinin-piperaquine; n/N – number of vivax recurrences/ number of people followed for that duration; NS – not stated; Pf – *P. falciparum*; Pv – *P. vivax*; SD – standard deviation;

References

171. Thimasarn K, Sirichaisinthop J, Chanyakhun P, Palananth C, Rooney W. A comparative study of artesunate and artemether in combination with mefloquine on multidrug resistant falciparum malaria in eastern Thailand. Southeast Asian J Trop Med Public Health. 1997;28(3):465-71. Epub 1998/04/30. PubMed PMID: 9561593.

172. Karbwang J, Na-Bangchang K, Thanavibul A, Mull R, Gathmann I. Dose-finding study of the efficacy of fixed-combination artemether/lumefantrine for the treatment of multidrug-resistant *Plasmodium falciparum* malaria in Thailand. Clin Drug Inv. 2000;19(5):343-8.

173. Kshirsagar NA, Gogtay NJ, Moorthy NS, Garg MR, Dalvi SS, Chogle AR, et al. A randomized, double-blind, parallel-group, comparative safety, and efficacy trial of oral co-artemether versus oral chloroquine in the treatment of acute uncomplicated *Plasmodium falciparum* malaria in adults in India. Am J Trop Med Hyg. 2000;62(3):402-8. Epub 2000/10/19. PubMed PMID: 11037786.

174. Denis MB, Davis TM, Hewitt S, Incardona S, Nimol K, Fandeur T, et al. Efficacy and safety of dihydroartemisinin-piperaquine (Artekin) in Cambodian children and adults with uncomplicated falciparum malaria. Clin Infect Dis. 2002;35(12):1469-76. Epub 2002/12/10. doi: 10.1086/344647. PubMed PMID: 12471565.

175. Stohrer JM, Dittrich S, Thongpaseuth V, Vanisaveth V, Phetsouvanh R, Phompida S, et al. Therapeutic efficacy of artemether-lumefantrine and artesunate-mefloquine for treatment of uncomplicated *Plasmodium falciparum* malaria in Luang Namtha Province, Lao People's Democratic Republic. Trop Med Int Health. 2004;9(11):1175-83. Epub 2004/11/19. doi: 10.1111/j.1365-3156.2004.01320.x. PubMed PMID: 15548313.

176. Hutagalung R, Paiphun L, Ashley EA, McGready R, Brockman A, Thwai KL, et al. A randomized trial of artemether-lumefantrine versus mefloquine-artesunate for the treatment of uncomplicated multi-drug resistant *Plasmodium falciparum* on the western border of Thailand. Malar J. 2005;4:46. Epub 2005/09/24. doi: 10.1186/1475-2875-4-46. PubMed PMID: 16179089; PubMed Central PMCID: PMCPMC1261533.

177. Denis MB, Tsuyuoka R, Poravuth Y, Narann TS, Seila S, Lim C, et al. Surveillance of the efficacy of artesunate and mefloquine combination for the treatment of uncomplicated falciparum malaria in Cambodia. Trop Med Int Health. 2006;11(9):1360-6. Epub 2006/08/26. doi: 10.1111/j.1365-3156.2006.01690.x. PubMed PMID: 16930257.

178. Denis MB, Tsuyuoka R, Lim P, Lindegardh N, Yi P, Top SN, et al. Efficacy of artemether-lumefantrine for the treatment of uncomplicated falciparum malaria in northwest Cambodia. Trop Med Int Health. 2006;11(12):1800-7. Epub 2006/12/21. doi: 10.1111/j.1365-3156.2006.01739.x. PubMed PMID: 17176344.

179. Thapa S, Hollander J, Linehan M, Cox-Singh J, Bista MB, Thakur GD, et al. Comparison of artemether-lumefantrine with sulfadoxine-pyrimethamine for the treatment of uncomplicated falciparum malaria in eastern Nepal. Am J Trop Med Hyg. 2007;77(3):423-30. Epub 2007/09/11. PubMed PMID: 17827354.

180. Rogers WO, Sem R, Tero T, Chim P, Lim P, Muth S, et al. Failure of artesunate-mefloquine combination therapy for uncomplicated *Plasmodium falciparum* malaria in southern Cambodia. Malar J. 2009;8:10. Epub 2009/01/14. doi: 10.1186/1475-2875-8-10. PubMed PMID: 19138388; PubMed Central PMCID: PMCPMC2628668.

181. Assefa A, Kassa M, Tadese G, Mohamed H, Animut A, Mengesha T. Therapeutic efficacy of Artemether/Lumefantrine (Coartem(R)) against *Plasmodium falciparum* in Kersa, South West Ethiopia. Parasit Vectors. 2010;3(1):1. Epub 2010/01/07. doi: 10.1186/1756-3305-3-1. PubMed PMID: 20051120; PubMed Central PMCID: PMCPMC2881066.

182. Na-Bangchang K, Ruengweerayut R, Mahamad P, Ruengweerayut K, Chaijaroenkul W. Declining in efficacy of a three-day combination regimen of mefloquine-artesunate in a multi-drug resistance area along the Thai-Myanmar border. Malar J. 2010;9:273. Epub 2010/10/12. doi: 10.1186/1475-2875-9-273. PubMed PMID: 20929590; PubMed Central PMCID: PMCPMC2959072.

183. Eshetu T, Abdo N, Bedru KH, Fekadu S, Wieser A, Pritsch M, et al. Open-label trial with artemether-lumefantrine against uncomplicated *Plasmodium falciparum* malaria three years after its broad introduction in Jimma Zone, Ethiopia. Malar J. 2012;11:240. Epub 2012/07/25. doi: 10.1186/1475-2875-11-240. PubMed PMID: 22824059; PubMed Central PMCID: PMCPMC3438107.

184. Leang R, Barrette A, Bouth DM, Menard D, Abdur R, Duong S, et al. Efficacy of dihydroartemisinin-piperaquine for treatment of uncomplicated *Plasmodium falciparum* and *Plasmodium vivax* in Cambodia, 2008 to 2010. Antimicrob Agents Chemother. 2013;57(2):818-26. Epub 2012/12/05. doi: 10.1128/AAC.00686-12. PubMed PMID: 23208711; PubMed Central PMCID: PMCPMC3553743.

185. Valecha N, Srivastava B, Dubhashi NG, Rao BH, Kumar A, Ghosh SK, et al. Safety, efficacy and population pharmacokinetics of fixed-dose combination of artesunate-mefloquine in the treatment of acute uncomplicated *Plasmodium falciparum* malaria in India. J Vector Borne Dis. 2013;50(4):258-64. Epub 2014/02/07. PubMed PMID: 24499847.

186. Lon C, Manning JE, Vanachayangkul P, So M, Sea D, Se Y, et al. Efficacy of two versus three-day regimens of dihydroartemisinin-piperaquine for uncomplicated malaria in military personnel in northern Cambodia: an open-label randomized trial. PLoS One. 2014;9(3):e93138. Epub 2014/03/29. doi: 10.1371/journal.pone.0093138. PubMed PMID: 24667662; PubMed Central PMCID: PMCPMC3965521.

187. Saunders DL, Vanachayangkul P, Lon C, Program USAMMR, National Center for Parasitology E, Malaria C, et al. Dihydroartemisinin-piperaquine failure in Cambodia. N Engl J Med. 2014;371(5):484-5. Epub 2014/07/31. doi: 10.1056/NEJMc1403007. PubMed PMID: 25075853.

188. Ebstie YA, Zeynudin A, Belachew T, Desalegn Z, Suleman S. Assessment of therapeutic efficacy and safety of artemether-lumefantrine (Coartem(R)) in the treatment of uncomplicated *Plasmodium falciparum* malaria patients in Bahir Dar district, Northwest Ethiopia: an observational cohort study. Malar J. 2015;14:236. Epub 2015/06/06. doi: 10.1186/s12936-015-0744-x. PubMed PMID: 26045199; PubMed Central PMCID: PMCPMC4464854.

189. Mekonnen SK, Medhin G, Berhe N, Clouse RM, Aseffa A. Efficacy of artemether-lumefantrine therapy for the treatment of uncomplicated *Plasmodium falciparum* malaria in Southwestern Ethiopia. Malar J. 2015;14:317. Epub 2015/08/15. doi: 10.1186/s12936-015-0826-9. PubMed PMID: 26271736; PubMed Central PMCID: PMCPMC4536736.

190. Wudneh F, Assefa A, Nega D, Mohammed H, Solomon H, Kebede T, et al. Open-label trial on efficacy of artemether/lumefantrine against the uncomplicated *Plasmodium falciparum* malaria in Metema district, Northwestern Ethiopia. Ther Clin Risk Manag. 2016;12:1293-300. Epub 2016/09/08. doi: 10.2147/TCRM.S113603. PubMed PMID: 27601913; PubMed Central PMCID: PMCPMC5005000.

191. Teklemariam M, Assefa A, Kassa M, Mohammed H, Mamo H. Therapeutic efficacy of artemether-lumefantrine against uncomplicated *Plasmodium falciparum* malaria in a high-transmission area in northwest Ethiopia. PLoS One. 2017;12(4):e0176004. Epub 2017/04/27. doi: 10.1371/journal.pone.0176004. PubMed PMID: 28445503; PubMed Central PMCID: PMCPMC5405980.

192. Itoh M, Negreiros do Valle S, Farias S, Holanda de Souza TM, Rachid Viana GM, Lucchi N, et al. Efficacy of Artemether-Lumefantrine for Uncomplicated *Plasmodium falciparum* Malaria in Cruzeiro do Sul, Brazil, 2016. Am J Trop Med Hyg. 2018;98(1):88-94. Epub 2017/11/17. doi: 10.4269/ajtmh.17-0623. PubMed PMID: 29141762; PubMed Central PMCID: PMCPMC5928740.

193. Wilairatana P, Krudsood S, Chokejindachai W, Bussaratid V, Silachamroon U, Viriyavejakul P, et al. A clinical trial of combination of artesunate and mefloquine in the treatment of acute uncomplicated falciparum malaria: a short and practical regimen. Southeast Asian J Trop Med Public Health. 1998;29(4):696-701. Epub 2000/04/20. PubMed PMID: 10772548.

194. Lefevre G, Looareesuwan S, Treeprasertsuk S, Krudsood S, Silachamroon U, Gathmann I, et al. A *clinical and pharmacokinetic trial of six doses of artemether-lumefantrine for multidrug-resistant Plasmodium* falciparum malaria in Thailand. Am J Trop Med Hyg. 2001;64(5-6):247-56. Epub 2001/07/21. PubMed PMID: 11463111.

195. Durrani N, Leslie T, Rahim S, Graham K, Ahmad F, Rowland M. Efficacy of combination therapy with artesunate plus amodiaquine compared to monotherapy with chloroquine, amodiaquine or sulfadoxine-pyrimethamine for treatment of uncomplicated *Plasmodium falciparum* in Afghanistan. Trop Med Int Health. 2005;10(6):521-9. Epub 2005/06/09. doi: 10.1111/j.1365-3156.2005.01429.x. PubMed PMID: 15941414.
