# Supplementary material for: The risk of Plasmodium vivax parasitaemia after P. falciparum malaria: An individual patient data meta-analysis from the WorldWide Antimalarial Resistance Network
Source: PLoS Med. 2020 Nov 19;17(11):e1003393. doi: 10.1371/journal.pmed.1003393 (PMC7676739; doi:10.1371/journal.pmed.1003393)
Supplement: S5 Table — (PDF) [file pmed.1003393.s013.pdf]

S5 Table. Comparison of baseline characteristics from included and targeted studies

Characteristic	Included studies (n=42)	Targeted studies* (n=25)
Region		
Asia-Pacific, studies (%)	39 (92.9)	18 (72.0)
Africa, studies (%)	1 (2.4)	6 (24.0)
The Americas, studies (%)	2 (4.8)	1 (4.0)
Year of enrolment†‡		
Pre-2009, studies (%)	31 (75.6)	15 (60.0)
2009-2017, studies (%)	10 (24.4)	10 (40.0)
Age (years), median (IQR)	17.0 (9.0, 29.0)	23.4 (17.1, 24.7)§
Male, % of patients	63.3	68.0¶

*Age and male percentage of targeted studies frequency weighted according to number of patients treated with artemisinin-based combination therapy; † Year of enrolment defined as the year study enrolment completed; ‡ Year of enrolment not available from one included study; § Mean age not available from nine studies; ¶ Percentage based on frequency weighted median, percentage not available from five studies
