# Supplementary material for: The risk of Plasmodium vivax parasitaemia after P. falciparum malaria: An individual patient data meta-analysis from the WorldWide Antimalarial Resistance Network
Source: PLoS Med. 2020 Nov 19;17(11):e1003393. doi: 10.1371/journal.pmed.1003393 (PMC7676739; doi:10.1371/journal.pmed.1003393)
Supplement: S6 Table — ACT, artemisinin-based combination therapy (PDF) [file pmed.1003393.s014.pdf]

**S6 Table. Prediction intervals for risk of *P. vivax* parasitaemia following artemisinin-based combination therapies**

| Day of follow up | ACT | Prediction interval (%)* |
|---|---|---|
| *Day 28* | | |
| | AL | 0 – 49.9 |
| | AA | 0 – 18.9 |
| | AM | 0 – 13.2 |
| | DP | 0 – 11.9 |
| *Day 42* | | |
| | AL | 0 – 74.4 |
| | AA | 0 – 52.9 |
| | AM | 0 – 35.9 |
| | DP | 0 – 28.0 |
| *Day 63* | | |
| | AL | # |
| | AA | # |
| | AM | 0 – 92.1 |
| | DP | 0 – 70.6 |

AA – artesunate-amodiaquine; ACT – artemisinin-based combination therapy; AL – artemether-lumefantrine; AM – artesunate-mefloquine; CI – confidence interval; DP – dihydroartemisinin-piperaquine; * Prediction interval for the risk of *P. vivax* parasitaemia by the specified follow up day.[17] Intervals were calculated from estimates of cumulative risk and standard errors generated from Kaplan-Meier survival analyses of individual patient data with estimates of between study variance derived from a random effects meta-analysis of study-level recurrence data using the Freeman-Tukey double arcsine transformation without adjustment to observed values[197]; # Prediction interval unable to be calculated as less than 3 estimates.

## References

17. Riley RD, Higgins JP, Deeks JJ. Interpretation of random effects meta-analyses. BMJ. 2011;342:d549. Epub 2011/02/12. doi: 10.1136/bmj.d549. PubMed PMID: 21310794.

197. Freeman MF, Tukey JW. Transformations related to the angular and the square root. Ann Maths Stats. 1950;21(4):607-11.
