# Supplementary material for: The risk of Plasmodium vivax parasitaemia after P. falciparum malaria: An individual patient data meta-analysis from the WorldWide Antimalarial Resistance Network
Source: PLoS Med. 2020 Nov 19;17(11):e1003393. doi: 10.1371/journal.pmed.1003393 (PMC7676739; doi:10.1371/journal.pmed.1003393)
Supplement: S7 Table — (PDF) [file pmed.1003393.s015.pdf]

S7 Table. Sensitivity analysis for associations between patient characteristics and rate of *P. vivax* parasitaemia between day 7 to 42 for the general model

Variable	Range of HR	Coefficient of Variation (%)
Gender		
Male	1.20-1.30	1.10
Female	1	-
Age category, years		
<5	2.84-3.58	2.97
5 to <15	1.61-1.99	1.97
≥15	1	-
Relapse periodicity		
Short	4.96-8.62	7.13
Long	1	-
P. falciparum gametocytes		
Yes	1.30-1.56	1.94
No	1	-
Mixed infection		
Yes	2.11-2.38	1.55
No	1	-
Parasitaemia, parasites per µL		
>100,000	1.44-1.67	1.64
≤100,000	1	-
Baseline haemoglobin (per 1 g/dL increase)	0.92-0.95	0.34
Drug		
AL	5.94-6.62	1.76
AA	1.90-2.56	2.86
AM	1.34-1.50	1.53
DP	1	-

AA – artesunate-amodiaquine; AL – artemether-lumefantrine; AM – artesunate-mefloquine; DP – dihydroartemisinin-piperaquine; HR – hazard ratio
