# Supplementary material for: The risk of Plasmodium vivax parasitaemia after P. falciparum malaria: An individual patient data meta-analysis from the WorldWide Antimalarial Resistance Network
Source: PLoS Med. 2020 Nov 19;17(11):e1003393. doi: 10.1371/journal.pmed.1003393 (PMC7676739; doi:10.1371/journal.pmed.1003393)
Supplement: S8 Table — (PDF) [file pmed.1003393.s016.pdf]

S8 Table. Parasite clearance according to treatment

Treatment	Number of patients*	Cleared on day 1 n (%)	Cleared on day 2 n (%)	Cleared on day 3 or later n (%)
Artemether-lumefantrine	1625	664 (40.9)	763 (47.0)	198 (12.2)
Artesunate-amodiaquine	487	303 (62.2)	167 (34.3)	17 (3.5)
Artesunate-mefloquine	5028	2376 (47.3)	2211 (44.0)	421 (8.8)
Dihydroartemisinin-piperaquine	2067	917 (44.4)	875 (42.3)	275 (13.3)
Overall	9208	4260 (46.3)	4017 (43.7)	931 (10.1)

* Number of patients with day of clearance assessed
