# Supplementary material for: The risk of Plasmodium vivax parasitaemia after P. falciparum malaria: An individual patient data meta-analysis from the WorldWide Antimalarial Resistance Network
Source: PLoS Med. 2020 Nov 19;17(11):e1003393. doi: 10.1371/journal.pmed.1003393 (PMC7676739; doi:10.1371/journal.pmed.1003393)
Supplement: S9 Table — (PDF) [file pmed.1003393.s017.pdf]

S9 Table. Relationship between day of parasite clearance, patient characteristics and rate of *P. vivax* parasitaemia between day 7 and 63

	Total N (n)	Adjusted HR (95% CI)	p value
Parasites cleared			
Day 3 or later	678 (128)	1.82 (1.44 - 2.30)	<0.001
Day 2	2986 (457)	1.48 (1.27 - 1.73)	<0.001
Day 1	3624 (324)	Reference	-
Age, years			
<5	525 (85)	1.97 (1.51 - 2.56)	<0.001
5 to <15	2567 (383)	1.73 (1.48 - 2.01)	<0.001
≥15	4196 (441)	Reference	-
Gender			
Male	4431 (576)	1.21 (1.06 - 1.39)	0.006
Female	2857 (333)	Reference	-
Relapse periodicity			
Short	6334 (877)	2.09 (0.83 – 5.30)	0.119
Long	954 (32)	Reference	-
P. falciparum gametocytes present			
Yes	807 (122)	1.48 (1.17 - 1.87)	0.001
No	6481 (787)	Reference	-
Mixed infection at baseline			
Yes	682 (140)	2.57 (2.11 - 3.12)	<0.001
No	6606 (769)	Reference	-
High parasite count, >100,000 parasites/µL			
Yes	457 (96)	1.52 (1.22 - 1.90)	<0.001
No	6831 (813)	Reference	-
Baseline haemoglobin (per 1 g/dL increase)	7288 (909)	0.95 (0.92 – 0.99)	0.004
Drug			
AL	1320 (300)	4.28 (3.21 - 5.72)	<0.001
AA	368 (15)	2.39 (1.02 – 5.60)	0.046
AM	4212 (446)	1.13 (0.89 - 1.43)	0.330
DP	1388 (148)	Reference	-

AA – artesunate-amodiaquine; AL – artemether-lumefantrine; AM – artesunate-mefloquine; CI – confidence interval; DP – dihydroartemisinin-piperaquine; HR – hazard ratio; n – number of patients with *P. vivax* recurrence; N – total number of patients
