# Supplementary material for: The risk of Plasmodium vivax parasitaemia after P. falciparum malaria: An individual patient data meta-analysis from the WorldWide Antimalarial Resistance Network
Source: PLoS Med. 2020 Nov 19;17(11):e1003393. doi: 10.1371/journal.pmed.1003393 (PMC7676739; doi:10.1371/journal.pmed.1003393)
Supplement: S10 Table — (PDF) [file pmed.1003393.s018.pdf]

S10 Table. Sensitivity analysis for associations between patient characteristics and rate of *P. vivax* recurrence between day 7 to 63 for the model including day of parasite clearance

Variable	Range of HR	Coefficient of Variation (%)
Parasites cleared		
Day 3 or later	1.76-1.92	1.18
Day 2	1.43-1.57	1.14
Day 1	Reference	-
Age, years		
<5	1.73-2.13	2.63
5 to <15	1.53-1.86	1.87
≥15	Reference	-
Gender		
Male	1.16-1.25	0.94
Female	Reference	-
Relapse periodicity		
Short	1.62-4.78	16.29
Long	Reference	-
P. falciparum gametocytes present		
Yes	1.40-1.52	1.08
No	Reference	-
Mixed infection at baseline		
Yes	2.45-2.71	1.36
No	Reference	-
High parasite count, >100,000 parasites/µL		
Yes	1.41-1.61	1.62
No	Reference	-
Baseline haemoglobin (per 1 g/dL increase)	0.94-0.97	0.31
Drug		
AL	3.99-5.46	3.70
AA	0.41-2.96	11.06
AM	1.07-1.18	1.33
DP	Reference	-

AA – artesunate-amodiaquine; AL – artemether-lumefantrine; AM – artesunate-mefloquine; DP – dihydroartemisinin-piperaquine; HR – hazard ratio
