# Supplementary material for: The risk of Plasmodium vivax parasitaemia after P. falciparum malaria: An individual patient data meta-analysis from the WorldWide Antimalarial Resistance Network
Source: PLoS Med. 2020 Nov 19;17(11):e1003393. doi: 10.1371/journal.pmed.1003393 (PMC7676739; doi:10.1371/journal.pmed.1003393)
Supplement: S11 Table — (PDF) [file pmed.1003393.s019.pdf]

S11 Table. Studies with follow up for 42 days or longer included in analysis of site factors

	Artemether-lumefantrine	Artesunate-amodiaquine	Artesunate-mefloquine	Dihydroartemisinin-piperaquine
Studies	12	5	23	16
Patients	2562	449	7021	4639
Study sites	18	7	29	21
Countries	7	3	9	8
By study:				
Enroll children <5 years	9	3	19	12
Risk of *P. vivax* at day 42 (range)	0 - 63.8%	2.0 - 22.0%	0 – 21.2%	0 – 21.8%
