# Supplementary material for: The risk of Plasmodium vivax parasitaemia after P. falciparum malaria: An individual patient data meta-analysis from the WorldWide Antimalarial Resistance Network
Source: PLoS Med. 2020 Nov 19;17(11):e1003393. doi: 10.1371/journal.pmed.1003393 (PMC7676739; doi:10.1371/journal.pmed.1003393)
Supplement: S12 Table — AL, artemether-lumefantrine (PDF) [file pmed.1003393.s020.pdf]

S12 Table. Relationship between patient characteristics and study site malaria prevalence and rate of *P. vivax* parasitaemia between day 7 and 42 in patients treated with artemether-lumefantrine

	Total N (n)	Adjusted HR (95% CI)	p value
Age, years			
<5	330 (121)	2.46 (1.67 - 3.62)	<0.001
5 to <15	612 (158)	1.78 (1.40 - 2.25)	<0.001
≥15	1141 (159)	Reference	-
Gender			
Male	1301 (278)	1.25 (1.03 - 1.53)	0.028
Female	782 (160)	Reference	-
Mixed infection at baseline			
Yes	160 (63)	2.35 (1.77 - 3.12)	<0.001
No	1923 (375)	Reference	-
Parasitaemia, >100,000 parasites/µL			
Yes	165 (57)	1.43 (1.05 - 1.93)	0.022
No	1918 (318)	Reference	-
Baseline haemoglobin (per 1 g/dL increase)	2083 (438)	0.95 (0.91 – 0.99)	0.025
P. falciparum incidence (per 1 case increase per 1000 person years)	2083 (438)	0.99 (0.97 - 1.02)	0.634
P. vivax incidence (per 1 case increase per 1000 person years)	2083 (438)	1.00 (0.99 - 1.02)	0.629

Only includes studies with a minimum follow up of 42 days. Single study outside of South-East Asia with long relapse periodicity excluded as relapse periodicity correlated with prevalence. There was no interaction between *P. falciparum* and *P. vivax* incidence, and they remained non-significant when only one of these variables was included in the analysis; CI – confidence interval; HR – hazard ratio; n – number of patients with *P. vivax* recurrence; N – total number of patients
