# Supplementary material for: The risk of Plasmodium vivax parasitaemia after P. falciparum malaria: An individual patient data meta-analysis from the WorldWide Antimalarial Resistance Network
Source: PLoS Med. 2020 Nov 19;17(11):e1003393. doi: 10.1371/journal.pmed.1003393 (PMC7676739; doi:10.1371/journal.pmed.1003393)
Supplement: S13 Table — (PDF) [file pmed.1003393.s021.pdf]

S13 Table. Relationship between patient characteristics and study site malaria prevalence and rate of *P. vivax* parasitaemia between day 7 and 42 in patients treated with artesunate-amodiaquine

	**Total N (n)**	**Adjusted HR (95% CI)**	**p value**
Age, years			
<5	32 (10)	3.98 (1.47 – 10.79)	0.007
5 to <15	132 (25)	2.82 (1.28 – 6.21)	0.010
≥15	164 (11)	Reference	-
Gender			
Male	196 (32)	2.31 (1.21 – 4.41)	0.011
Female	132 (14)	Reference	-
Mixed infection at baseline			
Yes	42 (14)	2.27 (1.17 – 4.38)	0.015
No	286 (32)	Reference	-
Baseline haemoglobin (per 1 g/dL increase)	328 (46)	0.88 (0.76 – 1.02)	0.099
P. falciparum incidence (per 1 case increase per 1000 person years)	328 (46)	1.01 (0.99 - 1.02)	0.377
P. vivax incidence (per 1 case increase per 1000 person years)	328 (46)	0.99 (0.96 - 1.03)	0.705

Only includes studies with a minimum follow up of 42 days. Relapse periodicity excluded as only studies from short relapse periodicity regions included; High parasite count excluded as strongly correlated with *P. falciparum* incidence, age and baseline haemoglobin. There was no interaction between *P. falciparum* and *P. vivax* incidence, and they remained non-significant when only one of these variables was included in the analysis; CI – confidence interval; HR – hazard ratio; n – number of patients with *P. vivax* recurrence; N – total number of patients
