# Supplementary material for: The risk of Plasmodium vivax parasitaemia after P. falciparum malaria: An individual patient data meta-analysis from the WorldWide Antimalarial Resistance Network
Source: PLoS Med. 2020 Nov 19;17(11):e1003393. doi: 10.1371/journal.pmed.1003393 (PMC7676739; doi:10.1371/journal.pmed.1003393)
Supplement: S14 Table — (PDF) [file pmed.1003393.s022.pdf]

S14 Table. Relationship between patient characteristics and study site malaria prevalence and rate of *P. vivax* parasitaemia between day 7 and 42 in patients treated with artesunate-mefloquine

	Total N (n)	Adjusted HR (95% CI)	p value
Age, years			
<5	706 (112)	5.01 (3.59 – 7.01)	<0.001
5 to <15	2686 (203)	2.44 (1.86 – 3.21)	<0.001
≥15	2943 (92)	Reference	-
Gender			
Male	3709 (246)	1.24 (1.01 - 1.51)	0.039
Female	2626 (161)	Reference	-
Mixed infection at baseline			
Yes	615 (71)	2.12 (1.59 – 2.81)	<0.001
No	5720 (336)	Reference	-
Parasitaemia, >100,000 parasites/µL			
Yes	380 (29)	1.58 (1.06 – 2.34)	0.023
No	5955 (378)	Reference	-
Relapse periodicity			
Short	5836 (400)	3.98 (0.41 – 38.31)	0.232
Long	499 (7)	Reference	-
Baseline haemoglobin (per 1 g/dL increase)	6335 (407)	0.93 (0.88 – 0.98)	0.004
P. falciparum incidence (per 1 case increase per 1000 person years)	6335 (407)	1.00 (0.98 - 1.03)	0.804
P. vivax incidence (per 1 case increase per 1000 person years)	6335 (407)	1.01 (0.97 - 1.04)	0.682

Only includes studies with a minimum follow up of 42 days. There was no interaction between *P. falciparum* and *P. vivax* incidence, and they remained non-significant when only one of these variables was included in the analysis; CI – confidence interval; HR – hazard ratio; n – number of patients with *P. vivax* recurrence; N – total number of patients
