# Supplementary material for: The risk of Plasmodium vivax parasitaemia after P. falciparum malaria: An individual patient data meta-analysis from the WorldWide Antimalarial Resistance Network
Source: PLoS Med. 2020 Nov 19;17(11):e1003393. doi: 10.1371/journal.pmed.1003393 (PMC7676739; doi:10.1371/journal.pmed.1003393)
Supplement: S15 Table — (PDF) [file pmed.1003393.s023.pdf]

S15 Table. Relationship between patient characteristics and study site malaria prevalence and rate of *P. vivax* parasitaemia between day 7 and 42 in patients treated with dihydroartemisinin-piperaquine

	Total N (n)	Adjusted HR (95% CI)	p value
Age, years			
<5	358 (58)	3.66 (2.09 – 6.40)	<0.001
5 to <15	783 (37)	1.01 (0.61 – 1.68)	0.967
≥15	1446 (38)	Reference	-
Gender			
Male	1634 (75)	1.02 (0.72 - 1.44)	0.913
Female	953 (58)	Reference	-
Mixed infection at baseline			
Yes	155 (21)	3.21 (1.88 – 5.47)	<0.001
No	2432 (112)	Reference	-
Parasitaemia, >100,000 parasites/µL			
Yes	182 (13)	1.40 (0.72 – 2.74)	0.320
No	2405 (120)	Reference	-
Baseline haemoglobin (per 1 g/dL increase)	2587 (133)	0.86 (0.79 – 0.94)	<0.001
Relapse periodicity			
Short	2325 (132)	6.84 (0.50 – 92.69)	0.148
Long	262 (1)	Reference	-
P. falciparum incidence (per 1 case increase per 1000 person years)	2587 (133)	1.00 (0.98 - 1.03)	0.724
P. vivax incidence (per 1 case increase per 1000 person years)	2587 (133)	1.00 (0.98 - 1.02)	0.779

Only includes studies with a minimum follow up of 42 days. There was no interaction between *P. falciparum* and *P. vivax* incidence, and they remained non-significant when only one of these variables was included in the analysis; CI – confidence interval; HR – hazard ratio; n – number of patients with *P. vivax* recurrence; N – total number of patients
