# Supplementary material for: The risk of Plasmodium vivax parasitaemia after P. falciparum malaria: An individual patient data meta-analysis from the WorldWide Antimalarial Resistance Network
Source: PLoS Med. 2020 Nov 19;17(11):e1003393. doi: 10.1371/journal.pmed.1003393 (PMC7676739; doi:10.1371/journal.pmed.1003393)
Supplement: Attachment — Submitted filename: hossain.pdf [file pmed.1003393.s025.pdf]

This paper describes an individual patient data meta–analysis of risk of parasitaemia.

I was asked for a statistical report and I interpret that to include all aspects of the design and conduct of the study.

Points of detail

Page 8 Is it likely that stopping the search two years ago has produced loss of important studies? If so this is a limitation to mention somewhere.

Page 9 If this is an existing data–base of studies then I am not clear why studies had to report one episode of *P vivax* in the manuscript as opposed to in the data–base.

Page 10 Presumably an enrolment deviation means the patient should never have been recruited or does it cover other protocol deviations?

Page 12 The sentence starting Risk of *P vivax* seems to partly duplicate the preceding paragraph. If it refers to something else then I think we need to see which confounders and why it is reasonable to assume there are no study site effects. That does seem quite a strong assumption to me.

Page 16 The model for the fall in haemoglobin does not seem to be explained in the methods or outlined in detail here. Have I missed it?

Page 17 I am not sure why the increased risk following AL means that other treatments should not get the same analysis.

Page 18, Figure 6 Is there really no relationship here? Although visual impression is often deceptive they do appear weakly correlated. I agree the model in Supplementary Table 8 seems to show no effect but is that because the two predictors are correlated?

Page 34, Table 2 If you have continuous data for heamoglobin why not use it as such? Categorising an essentially continuous variable wastes information (Altman and Royston, 2006; Royston et al., 2006) and leads to models which are often implausible as they predict the effect remaining flat within categories and then jumping to a new value at the category boundary.

There is a leave–one–out sensitivity analysis promised on page 12 but I do not see it reported anywhere. Supplementary material would be a fine place

to put it.

When the authors refer to study site in the description of the frailty model I assume they mean the finest classification: sites within studies. If not then I think the model needs changing.

Point of more substance

The authors refer to the degree of heterogeneity, for instance on page 15, but have not quantified it at all.

In the presence of substantial heterogeneity Riley et al. (2011) recommend prediction intervals to show where a new study (or in this case perhaps a new study site) might fall. Confidence intervals tell us how precisely we have estimated the mean of th effects but is that the clinically relevant issue rather than estimating what might happen in a new environment?

Summary

Mostly requests for additional information and clarification.

Michael Dewey

References

D G Altman and P Royston. The cost of dichotomising continuous variables. *British Medical Journal*, 332:1080, 2006.

R D Riley, J P T Higgins, and J J Deeks. Interpretation of random effects meta–analyses. *British Medical Journal*, 342:964–967, 2011.

P Royston, D G Altman, and W Sauerbrei. Dichotomizing continuous predictors in multiple regression: a bad idea. *Statistics in Medicine*, 25:127–141, 2006.
