# Supplementary material for: The risk of Plasmodium vivax parasitaemia after P. falciparum malaria: An individual patient data meta-analysis from the WorldWide Antimalarial Resistance Network
Source: PLoS Med. 2020 Nov 19;17(11):e1003393. doi: 10.1371/journal.pmed.1003393 (PMC7676739; doi:10.1371/journal.pmed.1003393)
Supplement: Attachment — Submitted filename: Reviewer_Plos Med.docx [file pmed.1003393.s024.docx]

**Comments to editor**

**Strength**

1. A large number of participants (15,341) were enrolled.

2. Unpublished studies were also reviewed.

3. The study was registered in the International Prospective Register of Systematic Reviews (PROSPERO: CRD42018097400).

4. The main analysis is appropriate for publication

5. The study contained the high impact value on the health policy.

6. I think the results are generalized in Asian countries.

**Weak points**

1. Some analyses needs to be performed as suggested in Results number 2, as the mixed infection should be confirmed by the PCR method.

2. Less studies from Africa were included in the study.

3. The quality of each included study needs to be assessed.

4. Some information in the introduction section to reader needs to be added.

5. Author should concentrate on the sentence if it needs to be cited in case of using the previous works.

**My comments**

**Abstract section**

1. Please clarify and check for the Journal guideline for writing manuscript in the first person style (Our study aimed to…).

2. The rational to used patients with “uncomplicated falciparum malaria treated with artemisinin-based combination therapy” should be explained more, why you have to use this study participants?

3. The systematic review was ended at 5 January 2018. Now, it is 2020. This mean that there might be more case reports of patients with “uncomplicated falciparum malaria treated with artemisinin-based combination therapy” in the database from 2018 to 2020. It is beneficial for reader if you can add more publications to 2020

**Introduction section**

1. There should be some information on the cause of malaria diseases, the prevalence, etiology, etc. before introduce the project of malaria elimination. This will guide readers familiar with the general knowledge of malaria before going into the aim of the study.

2. There should be a reference in this sentence “Although the burden of malaria has fallen in many regions, global estimates of malaria cases have 144 plateaued and even risen in some areas over the past five years.”

3. There should be a reference in this sentence “Radical cure with 8-aminoquinoline compounds can reduce the risk of P. vivax relapse significantly, however, both primaquine and tafenoquine can cause haemolysis in individuals with glucose-6-phophate dehydrogenase (G6PD) deficiency.”

**Method**

**Search strategy and selection criteria**

1. As the study adhere the PRISMA guideline, I did not see the “Quality of the included studies” which assess the quality of each included study. The authors stated in the supplementary file (PRISMA, method, page 13 of the manuscript) bot not present in the manuscript.

2. Again, it is beneficial for reader if you can add more publications to 2020

3. The clarification of excluding the study must be stated in the manuscript. Why pregnant patients or patients with severe malaria were excluded from the study? Can we include and separate the analysis to “severe malaria” and “uncomplicated malaria” in the present study?

4. Why you exclude patients with hyperparasitaemic infection? Please clarify this in the manuscript.

**Data collection and definitions**

1. What is/are rational to define Day 7 haemoglobin? Do you have any reference?

2. What is/are rational to define the anemia (Hb less than 10 g/dL) ? Do you have any reference?

3. Why the short relapse periodicity was defined as a median time to relapse of <47 days?

**Statistical analysis**

I think the analysis is appropriate.

**Ethics**
The systematic review was not requiring the ethical approval but are there any ethical approval certificates for used the data from the WWARN? Please provide the evidence.

**Results**

1. Do author excluded “In two studies, 448 patients were coadministered a single dose of

298 primaquine.”? as it is the exclusion criteria.

2. Confirming the mixed infection of *P. falciparum* and *P. vivax* by the microscopy is depending on the expertized level of microscopists. Moreover, the sensitivity of the microscopy is low. Now we know that confirming the mixed infection should rely on the results from PCR analysis. How many studies used the microscopy and PCR to confirm mixed infection in the included studies? Can author subgroup analyses “the studies using the microscopy” and “the studies using the PCR”? This will make the results complexes and more interesting.

3. Lacking “the quality of the included studies”

**Discussion**

No comment
