# Supplementary material for: The risk of Plasmodium vivax parasitaemia after P. falciparum malaria: An individual patient data meta-analysis from the WorldWide Antimalarial Resistance Network
Source: PLoS Med. 2020 Nov 19;17(11):e1003393. doi: 10.1371/journal.pmed.1003393 (PMC7676739; doi:10.1371/journal.pmed.1003393)
Supplement: Attachment — Submitted filename: WWARN Pv after Pf_Response.docx [file pmed.1003393.s026.docx]

**Editorial Requests:**

1. ***Reviewer 1 queried whether first-person phrasing was accepted by the journal (eg, "our analysis"; "we did"), just to inform the authors, we absolutely do accept and often will prefer this type of writeup as it is more direct and succinct.**

Thank you. We have retained the first-person in our manuscript narrative.

2. **At this stage, we ask that you include a short, non-technical Author Summary of your research to make findings accessible to a wide audience that includes both scientists and non-scientists. The Author Summary should immediately follow the Abstract in your revised manuscript. This text is subject to editorial change and should be distinct from the scientific abstract.**

We have added a non-technical author summary after the abstract.

3. **In addition, we request that you upload any figures associated with your paper as individual TIF or EPS files with 300dpi resolution at resubmission; please read our figure guidelines for more information on our requirements: https://journals.plos.org/plosmedicine/s/figures. PACE helps ensure that figures meet PLOS requirements. To use PACE, you must first register as a user. Then, login and navigate to the UPLOAD tab, where you will find detailed instructions on how to use the tool.**

We have updated the figures as requested.

**Reviewer #1**

We thank the reviewer for their positive and helpful response.

**Abstract**

**1. Please clarify and check for the Journal guideline for writing manuscript in the first person style (Our study aimed to…).**

As per editor’s comments we have retained the narrative using the first-person.

**2. The rational to used patients with “uncomplicated falciparum malaria treated with artemisinin-based combination therapy” should be explained more, why you have to use this study participants?**

Artemisinin-based combination therapies (ACTs) are now the first line treatment of uncomplicated falciparum malaria as recommended by the WHO and almost all malaria endemic countries. Our study sought to define the risk of *P. vivax* after *P. falciparum* in this population. We have revised the Introduction to the Abstract to clarify this.

**3. The systematic review was ended at 5 January 2018. Now, it is 2020. This mean that there might be more case reports of patients with “uncomplicated falciparum malaria treated with artemisinin-based combination therapy” in the database from 2018 to 2020. It is beneficial for reader if you can add more publications to 2020**

The process of identifying relevant clinical trials, contacting investigators, mapping and uploading individual patient data, and the complete analysis is time consuming and we have had to draw time limits on the review process. Whilst it would desirable to add data from 2018 to 2020 to the analysis, this would take a considerable amount time (at least 12 months), during which even more trials will have been published and excluded. Our sample size is comprehensive for the 58 years of the study time period that is stated and it is already sufficiently powered for the proposed analysis. We would therefore prefer to leave the review within the stated time period.

We have added the lack of the most contemporaneous studies to our limitations in the discussion (Line 529).

*“Our study has a number of limitations. The systematic review included all P. falciparum clinical trials between 1960 and 2018, although the analysis was restricted to patients who were treated with an ACT which covers studies conducted between 1991 and 2018.”*

**Introduction section**

**1. There should be some information on the cause of malaria diseases, the prevalence, aetiology, etc. before introduce the project of malaria elimination. This will guide readers familiar with the general knowledge of malaria before going into the aim of the study.**

We have added a sentence presenting the global burden of malaria to the start of the introduction.

**2. There should be a reference in this sentence “Although the burden of malaria has fallen in many regions, global estimates of malaria cases have 144 plateaued and even risen in some areas over the past five years.”**

We have now referenced the 2019 WHO World Malaria Report at the end of this sentence.

**3. There should be a reference in this sentence “Radical cure with 8-aminoquinoline compounds can reduce the risk of P. vivax relapse significantly, however, both primaquine and tafenoquine can cause haemolysis in individuals with glucose-6-phophate dehydrogenase (G6PD) deficiency.”**

We have now referenced a systematic review on haemolytic risk following 8-aminoquinoline drugs.

**Method**

**1. As the study adhere the PRISMA guideline, I did not see the “Quality of the included studies” which assess the quality of each included study. The authors stated in the supplementary file (PRISMA, method, page 13 of the manuscript) bot not present in the manuscript.**

A risk of bias table has been added to the supplement (S1 Text).

**2. Again, it is beneficial for reader if you can add more publications to 2020**

As stated in the response to Abstract #3, we believe that this is would cause significant delays and would add little to the analysis.

**3. The clarification of excluding the study must be stated in the manuscript. Why pregnant patients or patients with severe malaria were excluded from the study? Can we include and separate the analysis to “severe malaria” and “uncomplicated malaria” in the present study?**

The rationale of the analysis was to explore the risks and benefits of providing primaquine radical cure to patients presenting with uncomplicated malaria. Whilst pregnant women and those presenting with severe malaria may also be at risk of *P. vivax* relapse, primaquine is not prescribed to these patients. It is contraindicated in pregnancy and there are other more urgent management issues to address in patients presenting with severe malaria. For this reason, these patients were excluded from both the original and current analysis.

We have now added a sentence to clarify this on Line 229: *“Since primaquine and tafenoquine are contraindicated in pregnant women and not recommended acutely in patients presenting with severe malaria, studies only enrolling these patients were also excluded from the analysis”.*

**4. Why you exclude patients with hyperparasitaemic infection? Please clarify this in the manuscript.**

We did not exclude all patients with hyperparasitaemia rather just the studies in which only patients with hyperparasitaemia were included, since these would give a skewed representation of the risk of *P. vivax* in patients with uncomplicated malaria. In the final analysis 1 study was excluded due to this criterion.

We have revised the text on line 241 to clarify this: *“Additional study-level exclusions were if studies only included travellers or soldiers or only patients with hyperparasitaemic infection, since these either reflected malaria acquired from diverse locations or a skewed representation of patients attending a clinic in a malaria endemic setting.”*

**Data collection and definitions**

**1. What is/are rational to define Day 7 haemoglobin? Do you have any reference?**

The nadir haemoglobin concentration usually occurs between day 3 and day 7 after starting antimalarial treatment. The most common observation to quantify the fall in Hb in antimalarial efficacy trials is usually undertaken on day 7.

We have added a reference for this on line 266:

*“The nadir haemoglobin following acute P. falciparum malaria occurs between day 3 and 7, with most studies measuring the first follow up haemoglobin 1 week after starting antimalarial treatment. The early decline and recovery of haemoglobin was therefore assessed from the haemoglobin measured between day 6 and 8 inclusive.”*

**2. What is/are rational to define the anemia (Hb less than 10 g/dL) ? Do you have any reference?**

Moderate anaemia is defined by the WHO as a haemoglobin <10 g/dL (WHO World Malaria Report 2019).

**3. Why the short relapse periodicity was defined as a median time to relapse of <47 days?**

As per the statistical analysis plan, long and short periodicity of relapses were classified according to the regions described by Battel et al (7), with short periodicity considered to include regions where the median periodicity was ≤42 days. Thus regions with the two highest periodicities (region 10 and 12) where the median periodicity is <47 days were categorised as “short” and others were categorised as “long”.

**Statistical analysis**

**The analysis is appropriate. Ethics The systematic review was not requiring the ethical approval but are there any ethical approval certificates for used the data from the WWARN? Please provide the evidence.**

We have provided the reference approval certificate from The Oxford Ethics in the supplementary information S1 Document

**Results**

**1. Do author excluded “In two studies, 448 patients were coadministered a single dose of primaquine.”? as it is the exclusion criteria.**

No, these patients were not excluded. A single dose of primaquine is given for its anti-gametocidal *P. falciparum* transmission blocking properties. At this dose it has no anti-hypnozonticidal activity. However, this statement is included for clarity of the patient population.

**2. Confirming the mixed infection of *P. falciparum* and *P. vivax* by the microscopy is depending on the expertized level of microscopists. Moreover, the sensitivity of the microscopy is low. Now we know that confirming the mixed infection should rely on the results from PCR analysis. How many studies used the microscopy and PCR to confirm mixed infection in the included studies? Can author subgroup analyses “the studies using the microscopy” and “the studies using the PCR”? This will make the results complexes and more interesting.**

We agree that patients with subpatent asexual *P. vivax* parasitaemia, and thus mixed species infection, may have been enrolled into the *P. falciparum* studies. Whilst PCR may have detected some of these infections the methods applied in clinical trials over the last 2 decades are highly variable and even if the data were available this would make a robust subgroup analysis problematic.

Reassuringly, previous PCR analyses have shown mixed infections to occur in less than 10% of patients presenting with uncomplicated malaria. ACTs are highly active against *P. vivax* and at a low level parasitaemia, clearance of the blood stage infection is almost guaranteed (>95% efficacy). Hence, conservative estimates suggest that *P. vivax* recrudescence would only account for 0.5% risk of *P. vivax* recurrences during follow up. Thus we do not believe that this would have changed our analysis or conclusions.

We have added this to the limitations in the discussion (line 505)

*“It is possible that patients with mixed infections of P. falciparum with low level P. vivax parasitaemia were misdiagnosed as P. falciparum mono-infections and included in the analysis. However the ACTs included in the analysis have high efficacy against P. vivax [63] and even if 10% of patients had had submicroscopic P. vivax at presentation, the risk of recrudescent P. vivax by day 63 would be no greater than 0.5%.”*

**3. Lacking “the quality of the included studies”**

We now present the quality of included studies in the supplementary file.

**Reviewer #2** Michael Dewey Statistical Reviewer

**1. Page 8 Is it likely that stopping the search two years ago has produced loss of important studies? If so this is a limitation to mention somewhere.**

Similar to Reviewer #1 comment.

We have added the lack of the most contemporaneous studies to our limitations in the discussion (Line 529).

**2. Page 9 If this is an existing database of studies then I am not clear why studies had to report one episode of *P vivax* in the manuscript as opposed to in the database.**

Antimalarial efficacy studies of *P. falciparum* are focused primarily on the risk of *P. falciparum* and often any episodes with *P. vivax* are not presented in the paper despite these events occurring and being documented. Furthermore, *P. vivax* recurrence is not a primary endpoint in *P.falciparum* clinical trials and data on such recurrences are not always available. However, to ensure potentially eligible studies were not missed, the existing WWARN database was then searched for additional cases where an episode of *P. vivax* was documented.

**3. Page 10 Presumably an enrolment deviation means the patient should never have been recruited or does it cover other protocol deviations?**

Yes – protocol deviations were patients enrolled in violation of each studies’ inclusion and exclusion criteria. Sometimes these are included in intention to treat analyses for RCTs, but we excluded these.

We have revised the text on line 260 to clarify this.

**4. Page 12 The sentence starting Risk of P vivax seems to partly duplicate the preceding paragraph. If it refers to something else then I think we need to see which confounders and why it is reasonable to assume there are no study site effects. That does seem quite a strong assumption to me.**

This sentence refers specifically to the Figures 4 and 5 and includes the same confounders in the preceding sentence. In view of the duplication we have deleted the text from the Methods and added as footnotes to both of these figures.

**5. Page 16 The model for the fall in haemoglobin does not seem to be explained in the methods or outlined in detail here. Have I missed it?**

We have revised the Methods (lines 305) to clarify this:

*“**To investigate the association between the change in haemoglobin and the rate of P. vivax parasitaemia, the absolute fall in haemoglobin between day 0 and day 7 was included in the multivariable Cox regression model.”*

**6. Page 17 I am not sure why the increased risk following AL means that other treatments should not get the same analysis.**

The risk was far greater following AL and less confounded by post treatment prophylaxis. However, we now present similar analyses for the other drugs and produce the scatter plots as supplementary figures S3 to S5.

**7. Page 18, Figure 6 Is there really no relationship here? Although visual impression is often deceptive they do appear weakly correlated. I agree the model in Supplementary Table 8 seems to show no effect but is that because the two predictors are correlated?**

We have repeated the analyses using the subnational incidences of malaria from MAP. The reviewer is correct that the risk of *P. vivax* following AL was correlated with the background incidence of Pv and Pf, however in the multivariable model this was no longer apparent. We have revised the text on line 443, and present the results for the other ACTs in Tables S10-S13.

*“The risk of P. vivax following AL was correlated with the site specific background incidence of P. vivax (r_s_=0.676, p=0.0029) and incidence of P. falciparum (r_s_=0.607, p=0.0098). However after controlling for confounding factors in a Cox regression model, neither of the background incidences were associated with the risk of P. vivax (AHR=0.99, 0.97 – 1.02; p=0.634 and AHR=1.00, 0.99 – 1.02; p=0.629 respectively, for every 1 case per annum per 1,000 population increase); Figure 6. The results were similar for the other ACTs; S10-13 Tables and S3-S5 Figures.”*

**8. Page 34, Table 2 If you have continuous data for haamoglobin why not use it as such? Categorising an essentially continuous variable wastes information (Altman and Royston, 2006; Royston et al., 2006) and leads to models which are often implausible as they predict the effect remaining at within categories and then jumping to a new value at the category boundary. There is a leave-one-out sensitivity analysis promised on page 12 but I do not see it reported anywhere. Supplementary material would be a fine place**

**to put it.**

We present Hb as both continuous and categorical variables in the univariable analysis, but used the dichotomous variable in the multivariable analysis, since this has greater clinical utility. We have now revised the text to use the continuous Hb in the multivariable analyses as requested.

The leave-one out sensitivity analysis is presented in the Supplementary material (S14 Table).

**9. When the authors refer to study site in the description of the frailty model I assume they mean the finest classification: sites within studies. If not then I think the model needs changing.**

Yes, this is correct. We used the finest classification in the frailty model.

**Point of more substance**

**10. The authors refer to the degree of heterogeneity, for instance on page 15, but have not quantified it at all. In the presence of substantial heterogeneity Riley et al. (2011) recommend prediction intervals to show where a new study (or in this case perhaps a new study site) might fall. Confidence intervals tell us how precisely we have estimated the mean of the effects but is that the clinically relevant issue rather than estimating what might happen in a new environment?**

We have calculated the prediction intervals as suggested to provide a clearer measure of the heterogeneity (S6 Table).

**Reviewer #3**

**This is a very well written paper on an important topic in malaria epidemiology that may have implications for Plasmodium vivax (Pv) elimination strategies. The study aims to define the risk factors of Pv parasitemia following a Pf infection in various settings using an individual-level meta-analysis. While this is a well done and interesting meta-analysis, there are number of comments the authors should address.**

**Major Comments**

**1. My main issue with the paper, as currently written, is a lack of explanation as to the rationale for the study. It is clear the authors hypothesis that a Pf infection, and subsequent treatment, increases the risk of a relapse from a concurrent hypnozoite Pv infection, but the authors offer little rationale as to why they believe this hypothesis to be true. Is it simply because in areas with both Pf and Pv transmission, individuals with a Pf infection would also be at greater risk for harboring a Pv infection because of myriad factors in low and heterogeneous transmission that put specific people at greater risk? Would it have to do with immunity? Declines in hemoglobin following Pf treatment are explored, but are not stipulated a priori as a potential reason for Pv reactivation. Such rationales for the primary hypothesis under study should be clearly stated in the introduction, tested in the methods and results, and then reassessed and interpreted in the discussion.**

Our primary agenda was to identify patient populations where there was potential benefit to extend the use of primaquine radical cure to patients presenting with *P. falciparum* monotherapy. Previous studies have shown high risks of *P. vivax* after *P. falciparum*, and these are much higher than one would expect for patients living in the same areas who have not have acute falciparum malaria (see Response to comment 2 below). Hence our hypothesis is that in coendemic areas, patients are exposed to both parasites and that a high proportion of those with *P. falciparum* will harbour asymptomatic *P. vivax* hypnozoites and as such may benefit from radical cure. Previously we have been cautious about whether acute *P. falciparum* malaria (due to fever or haemolysis) actually activates the hypnozoites, or whether the time merely reflects an independent process in patients with dormant liver stages. Since recurrent *P. vivax* from both of these scenarios indicate potential benefit of radical cure the actual mechanism is probably of secondary significance.

However, a critical result of our current analysis is the correlation between the risk of relapse and the early parasite response – those who were slow to clear their parasites had a 1.8 fold increased risk of relapse – as we state in the discussion we believe that this provides important evidence for *P. falciparum* activating the hypnozoites.

We have revised the discussion to offer greater explanation for the cause of the high risk of *P. vivax*, and dismiss the confounding of effect of reinfection (see Comment#2 below) and we now attribute these recurrences to relapses that have been triggered by acute falciparum malaria (Line 497) . We have also clarified the rationale for the study at the end of the introduction which also states that the likely explanation is reactivation of *P. vivax* hypnozoites.

**2. One of the major weakness of this study is disentangling what the level of Pv relapse means among individuals with a Pf infection that are treated with various ACTs (e.g. cumulative risk of Pv parasitemia were 3.7%, 10.2% and 22.2%, at days 28, 42 and 63 respectively - lines 322-324). But how does this compare to the underlying rate of Pv parasitemia/relapse at these time points among individuals without a Pf infection who never received treatment? I.e. - What is the additional risk of Pv relapse due to a Pf-treated infection compared to those without a Pf infection? I understand the authors do not have such data on individuals without a Pf infection, but that comparison is what is needed to actually test if the rate of relapse in Pv is higher than what would be expected among those without a Pf clearance. This limitation needs to be clearly stated and discussed. Can the authors offer any insight from the literature into how they think Pv parasitemia/activation levels would differ between those with and without a Pf infection and treatment?**

We agree that independent cohorts of non infected individuals in the same locations would have strengthened the conclusions of underlying coinfection considerably. Unfortunately within the context of clinical efficacy trials these data are almost never available. However, in coendemic countries a 10-40% risk of *P. vivax* within 63 days of *P. falciparum* is far higher than one would expect by chance in these populations. Using data from the Malaria Atlas Project, the estimated subnational incidence of *P. vivax* for the study sites included in the study were between 0.2 and 151 per 1000 person years. If one conservatively ignores any post treatment prophylaxis following antimalarial treatment and selects the worst case scenario (151 per 1000 person years in PNG), then the estimated maximum risk of a new *P. vivax* infection within 63 days would be 2.6%. Hence, irrespective of the underlying cause, the risk of *P. vivax* after *P. falciparum* is 5-21 fold higher than would be expected in the non *P. falciparum* infected population.

We have now revised the discussion to acknowledge this limitation and highlight the predicted background risk – Line 495:

*“We could not discriminate whether recurrences were due to relapse or reinfection in patients continuing to reside in a malaria endemic area.* *However, the subnational incidence of P. vivax infection in the study sites included in our study ranged from 0.2 to 151 per 1000 person years.{ Battle, 2019 #1703} If one conservatively assumes 2 weeks post-treatment prophylaxis following antimalarial treatment and the highest risk of reinfection at the worst case scenario of the included study sites, then the maximum risk of a new infection with P. vivax within 63 days of P. falciparum would be 2.0%. The risks of P. vivax parasitaemia in our analysis were thus at least 6- to 21-fold greater than could be explained by reinfection alone.”*

**3. Could one factor that might explain the potential increased risk of Pv relapse among individuals treated for a Pf infection be the underlying level of Pf vs Pf transmission risk in the study sites - perhaps measured as the ration of Pf:Pv infections at baseline? If areas with much more Pv transmission relative to Pf transmission had higher risk of Pv relapse as compared to areas with higher Pf to Pv transmission at specified follow-ups, to me this would indicate that those with a Pf infection are just at higher risk of any Plasmodium infection. Can the authors include a Pf:Pv ratio indicator, perhaps categorized, as a covariate in the Cox PH models assessing the risk factors of Pv parasitemia? Assuming this could be derived by MAP estimates of PfPR and PvPR for each study site?**

This raises an important aspect that we have tried to explore, using background information on malaria transmission. We have revisited the analysis using annually stratified incidence rates. However, our analyses still fail to provide useful insights. A key issue is that *P. falciparum* clinical trials tend to be conducted in areas with sufficient infection rates where it is feasible to enrol patients in a reasonable period of time. Whilst we acknowledge this limitation in the generalisability of our findings, ultimately the next stage of investigation will require prospective clinical trials to quantify where primaquine or tafenoquine radical cure are most beneficial and we state this clearly in the final paragraph of the discussion.

**4. Can the authors more clearly state in the discussion the major limitation of the study that they cannot distinguish between a new Pv infection and a reactivation of a previous infection? Are the levels of parasitemia after 28, 42 and 63 days in line with transmission risk for a new Pv infection over this period in the study sites? I doubt they are and this argument should be made that they are likely reactivations because they are much higher than transmission would likely allow for them to be new Pv infections over these relatively short time periods?**

This comment is similar to that outlined in #2 above. And we have now revised the discussion to highlight this and to show that new infections with *P. vivax* could not account for these findings.

**5. Did the Cox models also account for malaria transmission season? They should if they have not.**

Whilst seasonality is an important factor in the incidence of *P. falciparum* in many locations, there is far less seasonality for *P. vivax* which is driven by relapses rather than reinfections. This is supported by a recent systematic review in which we have shown that at least 70% of recurrent *P. vivax* infections are due to relapsing infections (Commons, AJTMH 2020). Accounting for seasonality is further complicated by a lack of detailed understanding of seasonal variation for all geographic locations, and by differences between locations and differences in relapse periodicity and thus effect, between locations.

**6. I would think the underlying level of risk of transmission of Pf and Pv would be a significant risk factor (or potential confounder) when assessing the risk of a Pf infection treated with various ACTs. The authors appear to try and account for this with a covariate in the models for malaria parasite prevalence from MAP (lines 230-231). Why was this then categorized as low / not low at a cut-point of PfPR = 1.5%? As defined, this was not significant in the Cox models? Did they try other cut-points or categories? Why not just keep PfPR continuous - to me that would be the best option for accounting for this very important factor in the analyses.**

Previously we used the cut off of 1.5% since this equally divides the studies into high and low prevalence settings, i.e. the median value. However we have now reanalysed the data, to include the subnational incidence data and have used a continuous variable as suggested. We could not discern a significant relationship between background incidence and the risk of *P. vivax*.

**7. The authors state a date range of 1960 - 2018 was used for the systematic review of Pf treatment studies, taken for a previous study. This is a bit disingenuous as ACTS did not become available until well after 1960 (I would imagine in the 1990s at the earliest)? The authors should revise the date ranges for the systematic review accordingly, as well as provide the date ranges of the earliest and latest included studies in their description of the systematic review results.**

The original search was conducted to identify all antimalarial clinical trials since 1960 of which ACT trials were only a subset. We have now stated that range of years during which the included studies were undertaken.

Line 336: *“Of these, 51 (33.3%) studies enrolling 15,903 patients between 1991 and 2016, included treatment with the four major ACTs and were eligible for inclusion in this analysis.”*

**8. It would be helpful if the authors could state how the covariates in the Cox PH models were defined (e.g. those in lines 257 - 259). For example, was mixed infection categorized as yes/no (0/1). Was hemoglobin a continuous variable?**

We have described the definitions of the covariates in lines 257-281 and Table 2.

**9. For the analysis assessing the site-specific risk factors associated with risk of Pv following treatment (section starting on line 378), why was this limited to those treated with AL? Why not include all ACTs and just account for ACT type with a fixed effect covariate in the model?**

This is a similar query to Reviewer #2, point 6. We now present data on the other ACTs in the supplementary file. However, the numbers of events within each site were significantly lower due to the prolonged post-treatment prophylactic effect of all partner drugs other than lumefantrine.
